# The enigmatic nucleus of the marine dinoflagellate *Prorocentrum cordatum*

**DOI:** 10.1128/msphere.00038-23

**Published:** 2023-06-26

**Authors:** Jana Kalvelage, Lars Wöhlbrand, Robin-Alexander Schoon, Fiona-Marine Zink, Christina Correll, Jennifer Senkler, Holger Eubel, Mona Hoppenrath, Erhard Rhiel, Hans-Peter Braun, Michael Winklhofer, Andreas Klingl, Ralf Rabus

**Affiliations:** 1 General and Molecular Microbiology, Institute for Chemistry and Biology of the Marine Environment (ICBM), Carl von Ossietzky University of Oldenburg, Oldenburg, Germany; 2 Plant Development, Botany, Ludwig-Maximilians-Universität München, Planegg, Martinsried, Germany; 3 Plant Proteomics, Institute of Plant Genetics, Leibniz Universität Hannover, Hannover, Germany; 4 Marine Biodiversity Research, Institute of Biology and Environmental Sciences (IBU), Carl von Ossietzky University of Oldenburg, Oldenburg, Germany; 5 Senckenberg am Meer, German Centre for Marine Biodiversity Research (DZMB), Wilhelmshaven, Germany; 6 Planktology, Institute for Chemistry and Biology of the Marine Environment (ICBM), Carl von Ossietzky University of Oldenburg, Oldenburg, Germany; 7 Sensory Biology of Animals, Institute of Biology and Environmental Sciences (IBU), Carl von Ossietzky University of Oldenburg, Oldenburg, Germany; 8 Research Center Neurosensory Science, Carl von Ossietzky University of Oldenburg, Oldenburg, Germany; Clemson University, Clemson, South Carolina, USA

**Keywords:** nucleus, dinoflagellate, dinokaryon, *Prorocentrum cordatum*, FIB/SEM, proteomics, genomics, chromosomes, nuclear functions

## Abstract

The marine, bloom-forming dinoflagellate *Prorocentrum cordatum* CCMP 1329 (formerly *P. minimum*) has a genome atypical of eukaryotes, with a large size of ~4.15 Gbp, organized in plentiful, highly condensed chromosomes and packed in a dinoflagellate-specific nucleus (dinokaryon). Here, we apply microscopic and proteogenomic approaches to obtain new insights into this enigmatic nucleus of axenic *P. cordatum*. High-resolution focused ion beam/scanning electron microscopy analysis of the flattened nucleus revealed highest density of nuclear pores in the vicinity of the nucleolus, a total of 62 tightly packed chromosomes (~0.4–6.7 µm^3^), and interaction of several chromosomes with the nucleolus and other nuclear structures. A specific procedure for enriching intact nuclei was developed to enable proteomic analyses of soluble and membrane protein-enriched fractions. These were analyzed with geLC and shotgun approaches employing ion-trap and timsTOF (trapped-ion-mobility-spectrometry time-of-flight) mass spectrometers, respectively. This allowed identification of 4,052 proteins (39% of unknown function), out of which 418 were predicted to serve specific nuclear functions; additional 531 proteins of unknown function could be allocated to the nucleus. Compaction of DNA despite very low histone abundance could be accomplished by highly abundant major basic nuclear proteins (HCc2-like). Several nuclear processes including DNA replication/repair and RNA processing/splicing can be fairly well explained on the proteogenomic level. By contrast, transcription and composition of the nuclear pore complex remain largely elusive. One may speculate that the large group of potential nuclear proteins with currently unknown functions may serve yet to be explored functions in nuclear processes differing from those of typical eukaryotic cells.

**IMPORTANCE:**

Dinoflagellates form a highly diverse group of unicellular microalgae. They provide keystone species for the marine ecosystem and stand out among others by their very large, unusually organized genomes embedded in the nuclei markedly different from other eukaryotic cells. Functional insights into nuclear and other cell biological structures and processes of dinoflagellates have long been hampered by the paucity of available genomic sequences. The here studied cosmopolitan *P. cordatum* belongs to the harmful algal bloom-forming, marine dinoflagellates and has a recently *de novo* assembled genome. We present a detailed 3D reconstruction of the *P. cordatum* nucleus together with comprehensive proteogenomic insights into the protein equipment mastering the broad spectrum of nuclear processes. This study significantly advances our understanding of mechanisms and evolution of the conspicuous dinoflagellate cell biology.

## INTRODUCTION

Dinoflagellates are protists (unicellular eukaryotes) of microscopic size that possess unique cell biological features, comprise metabolically versatile keystone organisms in the marine carbon cycle, and occupy various habitats in marine and freshwater ecosystems. Dinoflagellates belong to the alveolates in the so-called TSAR (Telonemia, Stramenopiles, Alveolata, Rhizaria) clade, which harbors a major share in eukaryotic diversity (1) and was shaped by a remarkable evolutionary history (2, 3). Currently, ~2,500 extant dinoflagellate species (~300 genera) are classified with the majority representing free-living marine species (4, 5). Having long relied on morphological features and only recently being complemented by molecular phylogenetic approaches, the taxonomy of dinoflagellates is a continued scientific debate (6). The order Prorocentrales represents a monophyletic group within the core dinoflagellates (2, 7) and is long known to belong to the thecate (plate-bearing) dinoflagellates (6, 8, 9). The cellulosic theca of Prorocentrales taxa has a prorocentroid tabulation, which is characterized by two main large thecal plates, completed by small platelets in the periflagellar area (6, 10). Two morphologically typical dinoflagellate flagella are not associated with furrows, but arise apically from one flagellar pore in the periflagellar area, reflected in the term desmokont flagellation (6, 10). *Prorocentrum cordatum* (synonym *P. minimum*) (11) has an oval or cordiform to triangular shape, a size range of 10–24 µm in length, spines, and a theca, covered with small spines and thecal (likely trichocyst) pores (12, 13).

Dinoflagellates are cosmopolitan, abundant members of phytoplankton in marine and freshwater ecosystems (14), where they play multi-facetted ecophysiological roles and inhabit diverse niches (15). As key primary producers, they account for approximately 50% of the total carbon fixed by phytoplankton (16). About half of the known dinoflagellate species are photosynthetic, including autotrophs and mixotrophs (4). The majority of core dinoflagellates occur as free-living (some of them are bloom-forming) organisms, whereas some coexist as endosymbionts (e.g., reef-building corals) or parasites of protists or multicellular eukaryotes (17, 18). Some species contribute substantially to the formation of harmful algal blooms (HABs), which adversely affect surrounding organisms and ecosystems (19). *P. cordatum* is a common HAB-forming dinoflagellate and globally distributed in the marine realm from tropical and subtropical waters to temperate regions of the oceans (20). *P. cordatum* is highly adaptable to changing environmental conditions as it survives under low light and high nutrient stress (21), also performs mixotrophy, and feeds on algal prey at low inorganic nutrient availabilities (22).

From a cell biological perspective, dinoflagellates stand out by their unique nucleus, reflected in the term dinokaryon. An obvious feature is the unusually large genome: early estimates of mean chromosome numbers were 4–325 (32 for *P. cordatum*) (23) translating into genome sizes in the 2- to 3-digit Gbp range (24). A recent *de novo* assembly of the haploid genome of *P. cordatum* CCMP 1329 revealed a size of ∼4.15 Gbp harboring 85,849 protein encoding genes and a BUSCO protein recovery of 61.4% (25). Nuclear peculiarities of dinoflagellates further involve the presence of various modified DNA bases (26), a 10-fold lower protein-to-DNA ratio as typical for eukaryotes (27, 28), permanently condensed chromosomes in a “semi-crystalline state” (29), apparent lack of histones (30) contrasted by the presence of histone-like proteins (HLPs) and dinoflagellate viral nucleoproteins (DVNPs) (27, 31, 32), a nuclear envelope persisting throughout the whole cell cycle (33), and formation of extensive endomembrane networks during mitosis (34). Furthermore, the mechanisms involved in DNA compaction versus dynamics and spatio-temporal control of transcription are largely unknown at present (35, 36). Prompted by these nuclear peculiarities, Kubai and Ris (37) used electron microscopic serial sections to construct first 3D models focusing on the transformation of the nucleus together with its chromosomes during cell division of *Crypthecodinium cohnii* (synonym *Gyrodinium cohnii*) already in the late 1960s. Most recent reports on 3D models of nuclei and other subcellular structures in various microalgae rely on large-scale morphometric data, generated by optical microscopy, synchrotron topographies, or spectroscopic approaches (38 - 41).

Considering the environmental relevance and the unique cell biology of dinoflagellates, the present study focused on the enigmatic nucleus of *P. cordatum* CCMP 1329, a model of free-living dinoflagellates. We pursued the following two major aims. First, using microscopic approaches, the overall architecture of the nucleus including its envelope and chromosomal composition should be elucidated. Second, the repertoire of nuclear proteins responsible for the multiple and complex nuclear processes of *P. cordatum* should be investigated by specifically analyzing its recently determined genome combined with a detailed proteomic analysis of enriched nuclei.

## RESULTS AND DISCUSSION

### 3D reconstruction of the nucleus

**Fig 1:**
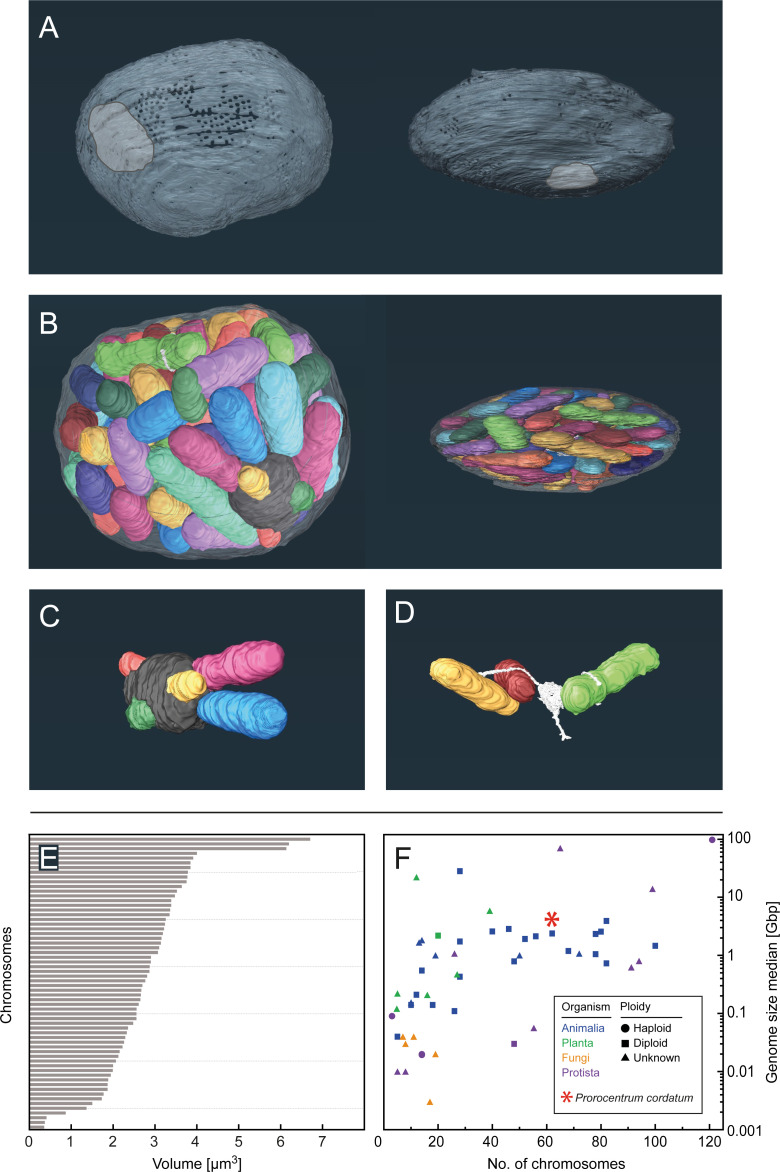
Three-dimensional reconstruction of the nucleus of *Prorocentrum cordatum* based on FIB/SEM images. (A) Distribution of nuclear pores across the nuclear envelope. Left panel, patch with high number of pores proximal to the nucleolus; right panel, pore-poor region (Movie S1). (B) Tight packing of chromosomes in the nucleus. Left panel, top view; right panel, side view. Chromosomes are arbitrarily colored, the nucleolus is marked dark gray and the nuclear membrane displayed transparently (Movie S2). (C) Focus on nucleolus with interacting chromosomes. (D) Conspicuous structure (white, probably extension of endoplasmic reticulum) interacting with several chromosomes. (E) Volume distribution of detected chromosomes. Further details are provided in Fig. S2 and Table S1. (F) Comparison of *P. cordatum* genome size and chromosome numbers with reported literature data. Further details and references are provided in Table S2.

The cell shape and subcellular structures of *P. cordatum* CCMP 1329 under the applied cultivation conditions agree well with previous reports (12, 13, 42, 43) and are displayed in Fig. S1. Thus, the here resolved nuclear 3D structures should be generally transferable. To study the 3D architecture of the nucleus of *P. cordatum*, embedded cells were subjected to focused ion beam (FIB)/scanning electron microscopy (SEM) (Fig. 1), followed by digital image analysis.

The double-layered nuclear envelope revealed that the overall shape of the nucleus follows the lens shape of the cell (Fig. 1A, right panel). Furthermore, the apertures of the nuclear pore complexes (NPCs) could be recognized. Notably, the NPCs are not randomly distributed across the nuclear envelope, but rather patch-like arranged in high numbers close to the nucleolus (Fig. 1A, left panel, Movie S1); only a few NPCs are scattered across the nuclear envelope. Such a spatial arrangement could streamline transport processes between the highly active nucleolus and the cytoplasm, and also might reflect a shielding of certain parts of the nuclear envelope due to local connectivity with the endoplasmic reticulum (44). This agrees with the observation of Wecke and Giesbrecht [cited in reference (45)] that the nuclear pores of *Prorocentrum* are arranged in closely packed hexagonal groups. We found the nuclear envelope to contain 475 NPCs, which greatly exceeds that of unicellular fungal microorganisms: *Saccharomyces cerevisiae* (budding yeast) was found to have 65–182 NPCs per cell (46) and *Schizosaccharomyces pombe* (fission yeast) 100–150 NPCs depending on the phase in the cell cycle (47). *P. cordatum* has a greater complexity in metabolism and cell structure compared to yeast, which apparently requires more nuclear pores to afford efficient nuclear import and export.

The chromosomes are tightly packed in the nucleus (Fig. 1B; Movie S2), which agrees with multiple observations previously reported for *P. cordatum* (48) and other dinoflagellates (10, 38, 40, 49). The nucleolus was found to interact with several chromosomes (Fig. 1C, marked in dark gray) in agreement with previous microscopic observations and it is assumed to function as location of ribosomal DNA transcription (50). The 2D structure of the nucleolus of *P. cordatum* revealed aggregated granular material of different densities (Fig. S2) and agrees well with previous reports from Dodge (42). A conspicuous structure was detected (Fig. 1D; Fig. S2, marked in white), which also interacts with some of the chromosomes. One may speculate that this structure represents parts of the endoplasmic reticulum extending into the nucleus and associated with membrane-bound mitotic channels as previously reported for *P. cordatum* (23, 51). In total, 62 chromosomes could be identified, which is in the range observed for other dinoflagellates (4–274) based on TEM image analysis (23) and genome sequencing (52, 53). It should be considered that the determined total number of chromosomes varies according to growth and developmental stage (54, 55).

**Fig 2:**
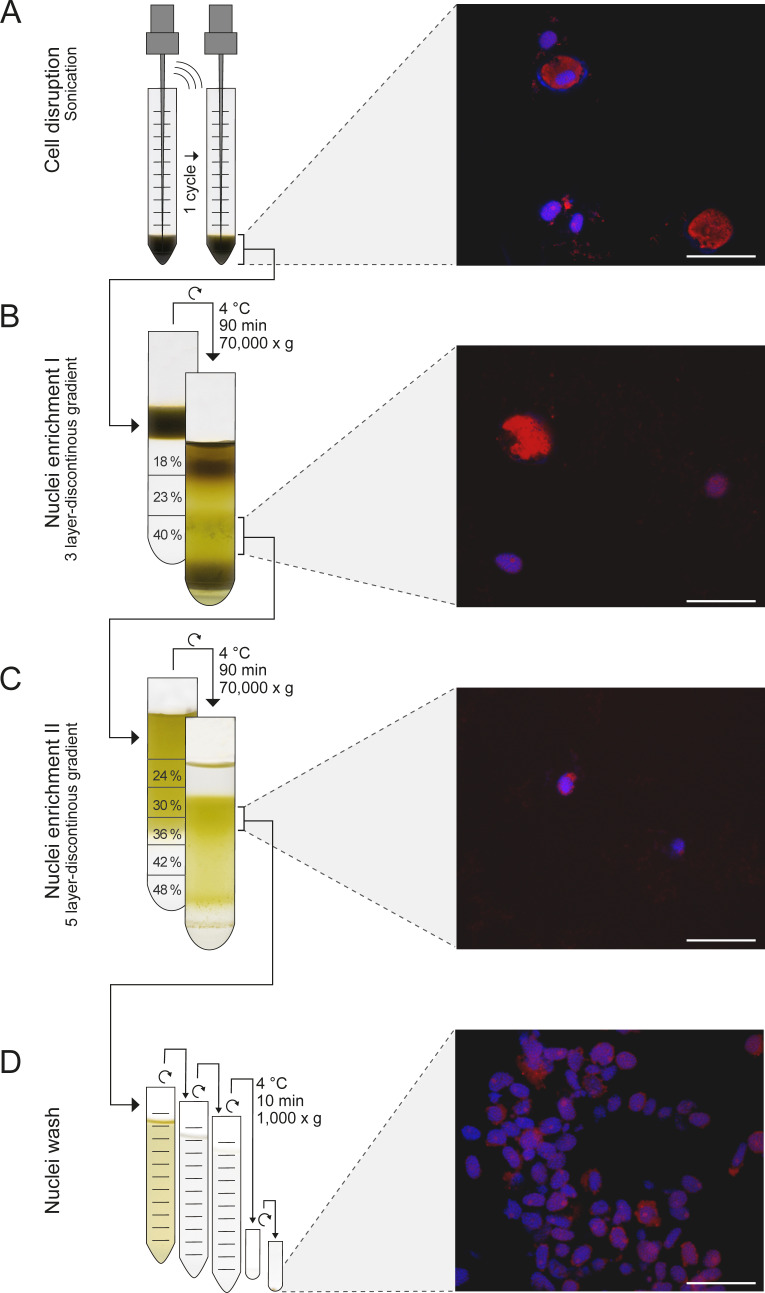
Procedure for nuclei enrichment from cells of *Prorocentrum cordatum*. (A) Gentle cell disruption via sonication. First (B) and second (C) enrichment steps involved 3- and 5-layer discontinuous Percoll-sucrose gradients, respectively (Percoll share given in percent). (D) Washing of nuclei in a sucrose/Tris buffer. Confocal laser scanning microscopic images, 63× magnification, 1.5× zoom. Staining of nucleic acids with 4',6'-diamidino-2-phenylindole (DAPI) (laser line, 405 nm; shutter intensity, 6%; detection, 415–480 nm); autofluorescence of pigments (laser line, 488 nm; shutter intensity, 35%; detection, 500–620 nm). Scale bar: A–D = 20 µm.

The reconstructed surfaces of these 62 chromosomes allowed us to estimate their volumes to range from 0.4 to 6.7 µm^3^ (on average 2.7 µm^3^) (Fig. 1E; for details, see Fig. S3), cumulatively accounting for ~80% of the ~217 µm^3^ nuclear interior. The numbers agree well with previous calculations reported for the dinoflagellate *Karenia papilionaceae* using synchrotron radiation-based hard X-ray tomography: ~273 µm^3^ nuclear interior with a chromosome-occupied volume of ~79% (40).

**Fig 3:**
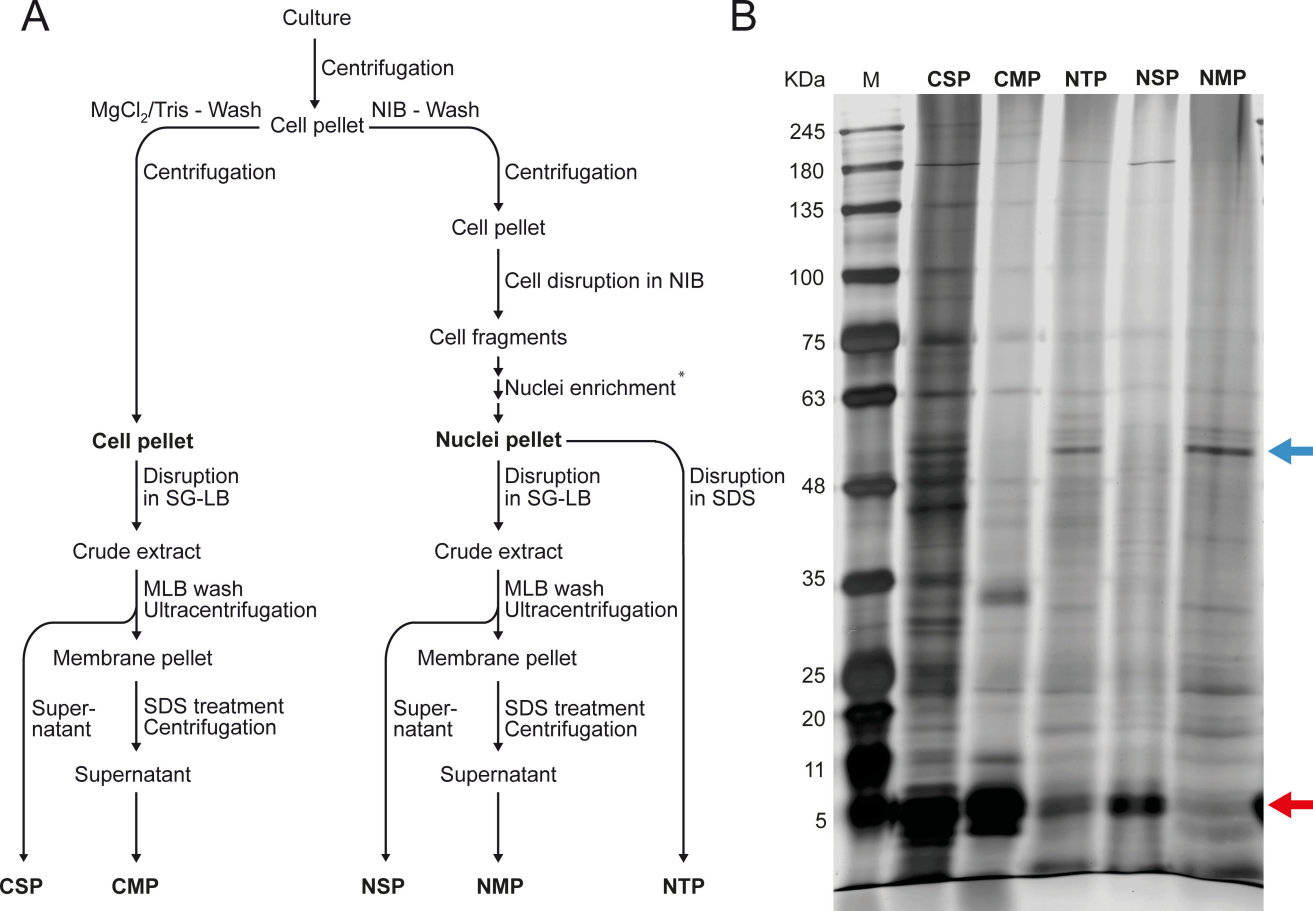
Subcellular fractionation of *Prorocentrum cordatum*. (A) Workflow for the differentiation of soluble and membrane protein-enriched fractions of entire cells *vs*. enriched nuclei. (B) Separation of prepared subcellular fractions via gradient SDS-PAGE (silver-stained). CMP, cellular membrane proteins; CSP, cellular soluble proteins; NMP, nuclear membrane proteins; NSP, nuclear soluble proteins; NTP, nuclear total protein.

Study of current literature revealed that *P. cordatum* belongs to the organisms with very large genomes organized in a high number of chromosomes (Fig. 1F; Table S1). Within the diverse group of dinoflagellates, the genome of the free-living *P. cordatum* represents an extremely large genome and is five to seven times larger than those of endosymbiotic *Symbiodinium microadriaticum* or *Breviolum minitum* (53, 56 - 58). Notably, the pure DNA volume [diploid ~4 Gbp, each bp taking 1 nm^3^; see Milo et al. (59)] is ~8 µm^3^ and comprises 3.7% of the nucleus, which is an estimate, since status of the cell cycle and chromosome duplication of the studied cell is unknown. To put this in perspective, a DNA volume fraction of at least 3.7% is comparable to that of spores of *S. cerevisiae* (59).

### Proteomics of enriched nuclei

#### Enrichment of nuclei

The robust theca of the *P. cordatum* cells presents a challenge for the preparation of intact nuclei. Therefore, a variety of previously reported preparation methods was tested (Table S2), e.g., enzymatic digestion of the cellulose-based theca (60, 61), different mechanical cell disruption approaches (62 - 64), diverse isolation buffers, and different separation techniques including filtration and various centrifugation gradients (62, 65 - 67). Since none of these methods yielded satisfactory results, the overall protocol was adapted to *P. cordatum* as schemed in Fig. 2. This procedure involves the following three main steps. First, cell disruption was achieved by washing cells in an ethanol/sucrose buffer [adapted from Levi-Setti et al. (68)] to partially disintegrate the cell envelope. This allowed gentle enough sonication to open the cells and at the same time preserve the intactness of the nuclear envelope (Fig. 2A). Second, enrichment of nuclei then involved ultracentrifugation with a three-layer Percoll-sucrose gradient as previously described for organelle enrichment in *Arabidopsis thaliana* (69). The nuclei-containing fraction was then further purified by another round of ultracentrifugation using a specifically designed five-layer Percoll-sucrose gradient (Fig. 2B and C). Third, the final fraction of enriched nuclei was washed with an adjusted Tris/sucrose buffer to further reduce the contaminant background (Fig. 2D). The efficiency of this procedure to enrich intact nuclei was assessed by fluorescence microscopy. A certain degree of carryover from other subcellular structures during this procedure of nuclei enrichment is comprehensible, considering the long-known connectivity of nuclei with the endoplasmic reticulum (ER) and the recently reported extensive reticular shape of chloroplasts and mitochondria in dinoflagellates [e.g., references (39, 41)].

#### Fractionation and electrophoretic separation

The enriched nuclei were used to prepare three different nuclear fractions: first, the nuclear total protein (NTP); second, the nuclear soluble protein (NSP); and third, the nuclear membrane protein (NMP) (Fig. 3A). For a detailed proteomic comparison, the soluble [cellular soluble proteins (CSP)] and membrane protein-enriched [cellular membrane proteins (CMP)] fractions (yielding fractions 4 and 5) of whole cells were also prepared. These five fractions were decomplexed by electrophoretic separation in large 1D gradient gels (Fig. 3B). Comparison of the different fractions revealed distinct patterns of cellular versus nuclear fractions as well as of soluble versus membrane fractions (both cell and nuclei). While distinct bands appeared enriched in the nuclei fractions (Fig. 3B, blue arrow), others are clearly depleted (Fig. 3B, red arrow), indicating successful preparation of nuclear protein-enriched fractions.

#### Mass spectrometric analyses and proteomic dataset

MS-based protein identification was based on two complementary approaches. First, per cellular/nuclear fraction, 34 gradient gel-separated sub-fractions were subjected to in-gel digest for comparing the different fractions facilitated by their gel-electrophoretic decomplexation (geLC dataset). Second, the soluble fractions (CSP and NSP) were directly subjected to in-solution digest, followed by in-depth analyses of the resultant complex lysates with a highly mass accurate MS instrument (shotgun dataset). The benefit of pre-electrophoretic fractionation applied in the geLC approach is evidenced by the high number of proteins identified specifically per fraction (Fig. S4B). Overall, the geLC approach yielded 819 versus 976 identified proteins for the whole cell versus the enriched nuclei, with a shared fraction of 388 proteins (Fig. S4Ci). Beyond that, the shotgun approach generated 2,897 versus 2,709 identified proteins for the CSP and NSP fractions with a common share of 1,993 proteins (Fig. S4Cii). Both approaches combined provided a total dataset of 4,052 different proteins with a slightly larger share contributed by the nuclear fractions (Fig. S4Ciii, iv), demonstrating enrichment of lower abundant (nuclear) proteins in the nuclear fractions.

**Fig 4:**
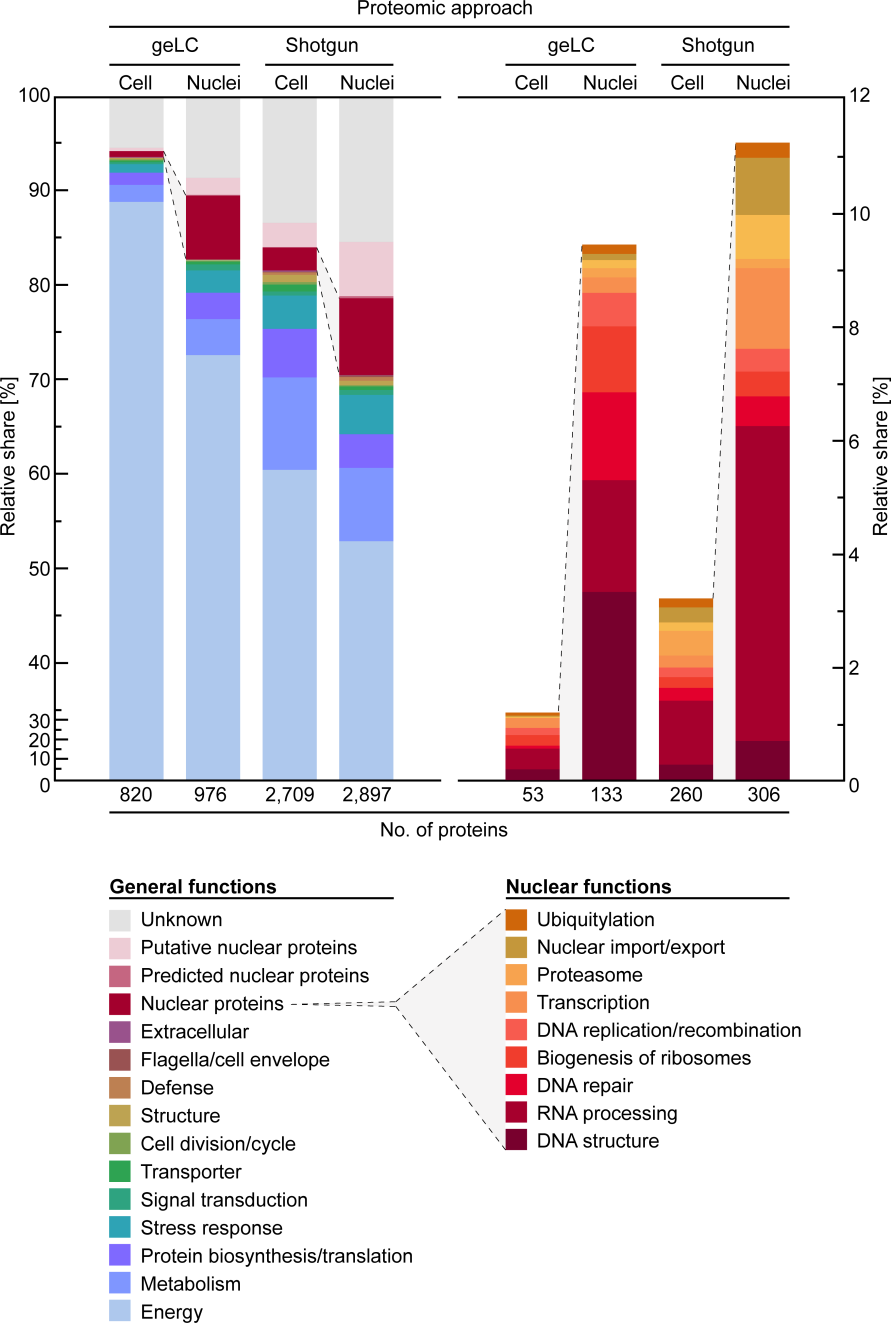
Functional categorization of proteins identified from *Prorocentrum cordatum*. Left panel, general functions; right panel, nuclear functions.

A specific and proteomics challenging feature of *P. cordatum* is the very high abundance of chloroplast-localized chromophore-binding proteins, as these constitute ~73% of the combined cellular dataset (geLC). This explains the detection of these proteins also in the enriched, combined nuclear fraction (geLC), where they account for ~51%. Moreover, their ease of MS-based detection (well-ionizable peptides and small protein size) hampers detection of lower abundant nuclear proteins (i.e., by competing for ionization and detection).

Although the number of proteins in the gel-based approach was notably lower, the differences between nuclear and cellular fractions were here oftentimes more pronounced.

#### Functional categorization

Multilayered bioinformatic functional prediction (56) combined with manual refinement allowed us to assign 2,455 out of 4,052 identified proteins to superordinate functional categories (remaining 1,597 proteins are of unknown function) (Fig. 4, left panel). Independent of the applied proteomic approach, a major share of identified proteins is affiliated with the categories energy, metabolism, and translation, underlining the importance of these processes. Note that aforementioned chromophore-binding proteins execute a certain bias in the energy and other categories.

Nuclear proteins (Fig. 4, left panel, red-brown segments) were up to ~12-fold enriched in the nuclear fraction (geLC) and many proteins of unknown function were found to be enriched in the nuclear fraction (up to 30%, shotgun; Fig. 4, left panel, gray segments). Indeed, for up to ~8.3% of these proteins, a nuclear localization could be corroborated by in-depth bioinformatic analyses (Fig. S4D), justifying their assignment as putative nuclear proteins (Fig. 4, left panel, light-pink segments).

Focusing on individual nuclear proteins (Fig. 4, right panel), most of them were found to be involved in RNA processing. In general, proteins of all nuclear functions appear to become similarly enriched in the nuclear fractions. Considering the distinct features of the *P. cordatum* nucleus and the current lack of functional understanding, the generated proteomic dataset, together with its recently sequenced genome, was mined to reconstruct nuclear functions.

### Nuclear processes and proteins

#### DNA condensation

Several studies predict models of compaction of dinoflagellate chromosomal DNA, suggesting their helicoidal arrangement in a liquid crystalline state, with a reduced amount of histones and the presence of substitute dinoflagellate-specific proteins (70 - 73). The large size of dinoflagellate genomes requires higher-than-average condensation of the DNA, which is even more demanding in view of the loss of bulk nucleosomal DNA packaging (2, 28). Although histones were long thought to be absent in dinoflagellates, they could be detected in previous studies, albeit at very low abundances and with divergent sequences compared to other counterparts in eukaryotes (74, 75).

The genome of *P. cordatum* encodes all four core proteins (H2A, H2B, H3, and H4), from which H2A, H2B, and H4 were detected in the nuclear protein datasets from geLC and shotgun analyses at very low level (Fig. 4, right panel). In view of the low abundances of histones in *P. cordatum* and other dinoflagellates, one can speculate that these proteins play only a subordinate function in DNA condensation (74, 75).

In addition to histones, dinoflagellates’ evolutionary history revealed that their nuclei have recruited other DNA-binding proteins, including DVNPs and bacterial HLPs (2). DVNPs are located to chromatin and show strong DNA-binding abilities, indicating their involvement in DNA compaction (31, 73). The genome of *P. cordatum* encodes 11 proteins homologous to functionally studied DVNP 5 of *Hematodinium* sp. (31), one of which was detected in very low abundance in the nuclear protein dataset (geLC). Since DNVPs could also be detected at comparably low amounts as histones, they probably have no major function in chromosome structuring in *P. cordatum*.

**Fig 5:**
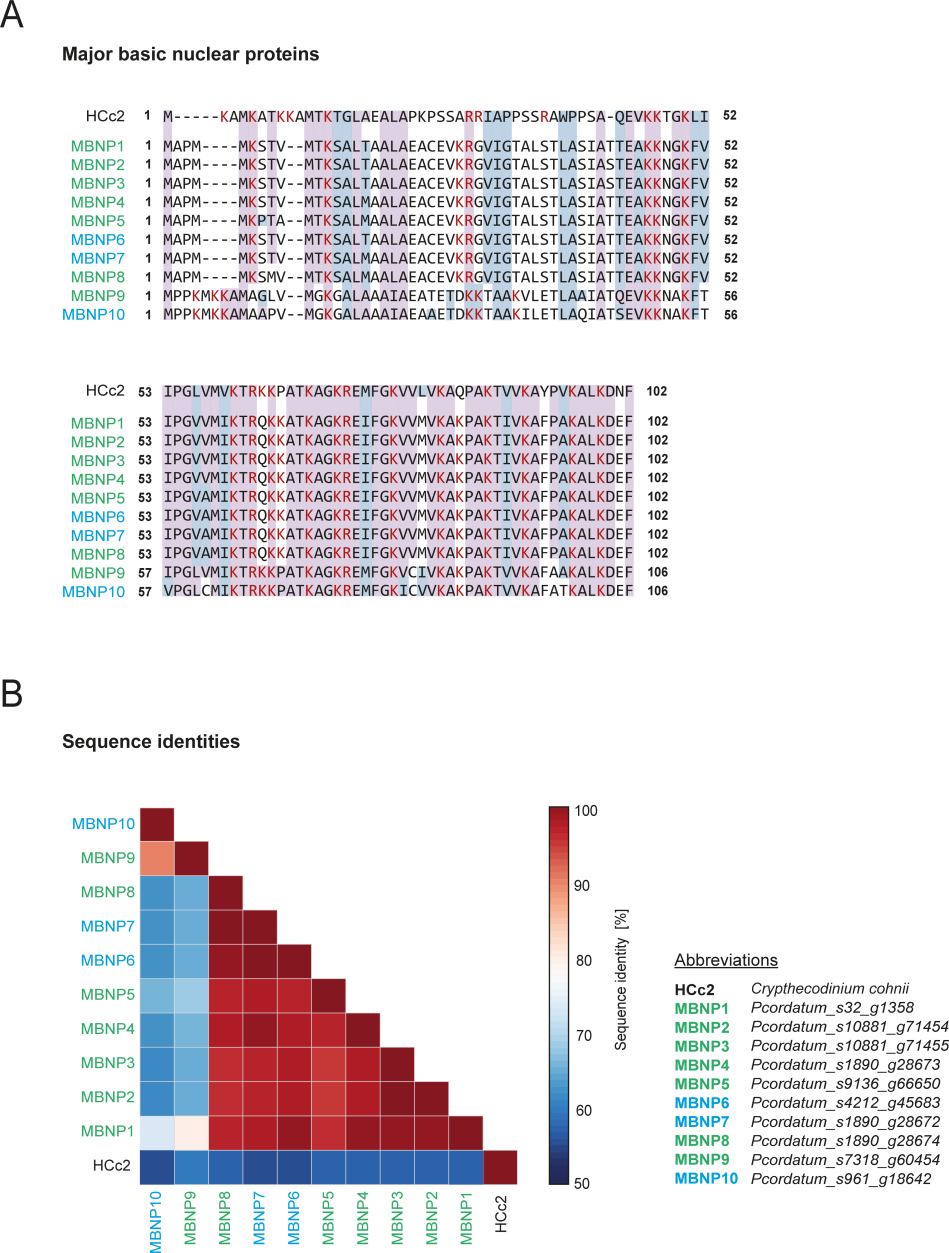
MBNPs of *Prorocentrum cordatum*. (A) Multiple alignment of MBNPs from *P. cordatum* compared to the HLP HCc2 from *Crypthecodinium cohnii*. (B) Pairwise comparison of sequence identities. Background coloring: purple, identical residues; gray-blue, conservative replacements; red-colored residues, basic amino acids lysine (**K**) and arginine (**R**).

Next to DVNPs, dinoflagellates have long been known to be rich in highly basic proteins bearing potential helix-turn-helix motifs for DNA-binding, e.g., in *Gymnodinium nelsonii, Lingulodinium polyedra,* and *Crypthecodinium cohnii* (27, 32, 76, 77). These so-called major basic nuclear proteins (MBNPs) differ completely from eukaryotic histones with respect to amino acid composition and by markedly lower DNA-binding ability (76). In *P. cordatum*, the MBNPs represented the most abundant species among the proteins assigned to nuclear functions within the geLC-analyzed nuclear fraction (Fig. 4, right panel), where it was identified ~29-fold more often than in the cellular fraction. This agrees with earlier reports, revealing MBNP transcripts to belong to the top 10 most abundant transcripts in dinoflagellates (78). The genome of *P. cordatum* harbors 11 genes encoding MBNPs, seven of which were identified. They display high sequence similarities (Fig. 5A and B) among each other (100–61%) and with the functional-studied HCc2 from *C. cohnii* (60–56%) (79). In contrast to DVNPs and histones, MBNPs could be detected in all analyzed protein fractions of *P. cordatum* and with a considerably higher abundance (geLC; ~137- and ~117-fold, respectively).

#### DNA replication

**Fig 6:**
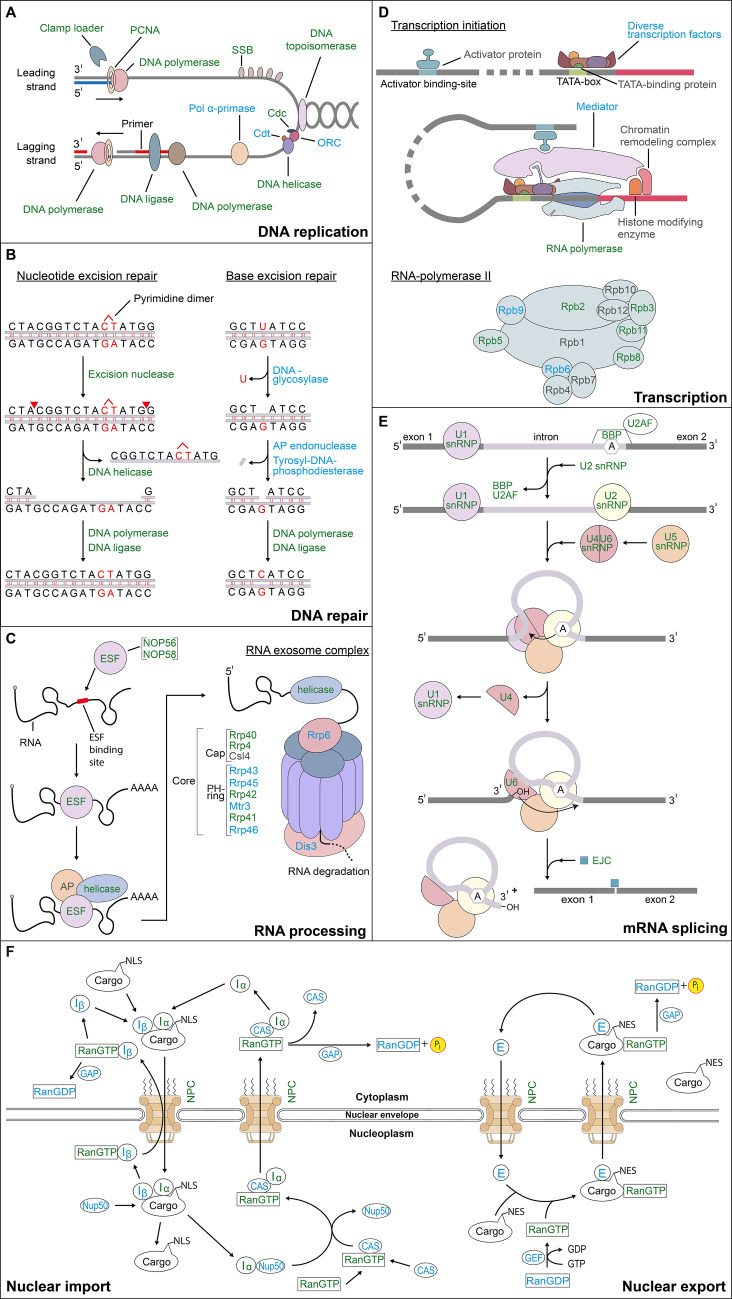
Reconstruction of nuclear processes in *Prorocentrum cordatum* based on the present proteomic dataset superimposed on general reported knowledge. Coloring: green, proteins identified; blue, proteins predicted only; gray, proteins not predicted. (A) DNA replication. Cdc6, cell division control protein; Cdt1, DNA replication licensing factor; PCNA, proliferating cellular nuclear antigen (sliding clamp); pol, polymerase; ORC, origin of replication complex; SSB, single-strand binding protein [adapted from reference (82) with permission of the publisher]. (B) DNA repair [adapted from reference (83) with permission of the publisher]. (C) RNA processing. AP, accessory proteins; Csl, exosome complex component Csl4; ESF, exosome-specificity factor; Mtr, exosome complex component mtR3; NOP, nucleolar protein; Rrp, rRNA biogenesis protein [adapted from reference (84) with permission of the publisher]. (D) Transcription. Rpb, RNA polymerase II subunit b [adapted from references (84, 83) with permission of the publisher]. (E) mRNA splicing. A, branch-point intron sequence; BBP, branch-point binding protein; EJC, exon junction complex; snRNP, small nuclear ribonucleoprotein; U2AF, U2 auxiliary factor [adapted from reference (83) with permission of the publisher]. (F) Nuclear import and export. CAS, CRISPR-associated proteins; E, exportin; GAP, GTPase-activating protein; GEF, guanine exchange factor; I, importin; NES, nuclear export signal; NLS, nuclear localization signal; NPC, nuclear pore complex; NUP, nucleoporin (NPC protein); RanGTP, ras-related nuclear protein; GTP, guanosine triphosphate [adapted from references (83, 84, 85) with permission of the publisher].

Most proteins involved in DNA replication (Fig. 6A) such as topoisomerase, DNA polymerase, DNA helicase, DNA ligase, single-strand binding proteins, cell division control proteins, PCNA (proliferating cell nuclear antigen) proteins, and replication factors of the clamp loader were identified in the proteome of *P. cordatum*. In particular, PCNA proteins were recently implicated to play a major role in DNA replication of dinoflagellates (80). This is corroborated by the present study as nine PCNA proteins were predicted from the genome, of which one was identified with higher abundance in the nuclear fractions (shotgun). Several components, such as DNA polymerase δ and ε, primase, and origin of replication complex, could only be predicted from the genome of *P. cordatum*. While DNA polymerase subunits were unexpectedly detected at only low abundances, a broad variety of viral RNA- and DNA-directed DNA polymerases were detected in the nuclear-enriched fractions at considerably higher abundances, indicating high impact of viral genome integrations in *P. cordatum* (81). Several known proteins of DNA replication were also identified in the dinoflagellate *Lingulodinum polyedra* (65).

#### DNA repair

Detected proteins of the well-known mechanisms of nucleotide/base excision repair (NER/BER) are schemed in Fig. 6B (86, 87). In case of NER, a candidate for an excision nuclease could be detected; the subsequent reactions are catalyzed by general enzymes (e.g., helicase), which were all detected. By contrast, for BER the initial specific enzymes (e.g., glycosylase and endonuclease) could only be predicted from the genome of *P. cordatum*.

#### RNA processing

A major share of nuclear proteins in *P. cordatum* could be assigned to mRNA and rRNA processing with a high number of proteins involved in multifunctional RNA quality control. Eleven subunits of the exosome complex (88) (Fig. 6C) were predicted from the genome with four of them identified in the proteomic dataset. Moreover, two exosome-specificity factors were identified from which one showed ~90-fold higher abundance. Further, various ATP-dependent DEAD-box RNA-helicases involved in RNA processing could be detected with ~11-fold higher abundance in the nuclear fraction (geLC).

#### Transcription

In view of transcriptional processes, dinoflagellates present various peculiarities compared to other eukaryotes. The canonical core promoter sequence (TATA-box/GC-box) seems to be absent in dinoflagellates (89). In *Lingulodinium* (as *Gonyaulax*) *polyedra* for example, the sequence CGTGAACGCAGTG was determined as possible transcriptional start site (89), while other studies revealed a TTTT-box instead (90). Remarkably, neither of these two promoter types could be detected in the genome of *P. cordatum*. Further, the TATA-box-binding protein (TBP) (91) is apparently not encoded in the genome of *P. cordatum*. In *C. cohnii*, a TBP-like factor presumably acts as promoter binding protein (92), which, however, could also not be predicted from the genome of *P. cordatum*. In total, only two transcriptional factors, TFIIB and TFIIH, could be predicted in the present study with TFIIH also identified at very low abundances in the cellular fraction (geLC). Thus, specimen-specific promoter regions and corresponding recognition mechanisms may occur among dinoflagellates.

The typical eukaryotic RNAP2 (Fig. 6D) consists of 12 subunits (93), which showed varying extent of genomic prediction and proteomic identification in *P. cordatum*: Rpb1/4/7/10/12 not predicted, Rpb6/9 predicted only, and Rpb2/3/5/8/11 identified (~4.5-fold higher abundance in the nuclear fraction (shotgun) (84). Among the non-predicted subunits, Rpb1 has a unique C-terminal domain (CTD), which consists of tandem repeats of the heptapeptide sequence “YSPTSPS” (94). The CTD is often phosphorylated at the serine residues to coordinate the localization of transcription and to recruit RNA processing factors to elongate the polymerase complex (94, 95). Notably, this common domain could not be detected for *P. cordatum*.

Studies on other dinoflagellates confirm the presence of multiple forms of DNA-dependent RNA polymerases as known for other eukaryotes, but probably with a reduced number of components and with different activity (35, 96). Functionality of the transcriptional machinery in eukaryotes usually requires several additional components. First, the spatial approximation of activator sites with RNAP2 is achieved by the mediator complex (~30 subunits) (97), which could, however, not be predicted from the genome of *P. cordatum* except for one subunit. Second, the chromatin remodeler (RSC) (six subunits) (98), which ensures DNA accessibility to transcription (98), could also not be predicted from the genome of *P. cordatum*. The absence of these two complexes in conjunction with the permanently condensed chromosome structure in dinoflagellates poses the question of how they achieve transcription. In *P. micans*, transcription is assumed to occur only on extrachromosomal DNA margins and not within the main body of a chromosome and the Z-type conformation of the DNA is to create possible sites for unwinding and DNA processing (99, 100).

Dinoflagellate-specific modes of transcriptional control are currently assumed to involve DNA and histone methylation. DNA methylation is discussed to enable transcription by modifying the chromatin structure (101) and to regulate gene expression (102). In accord with this assumption, DNA-cytosine-methyltransferases (DNMTs) could be detected at ~11-fold higher abundances in the nuclear fraction (geLC) of *P. cordatum*. Interestingly, a recent study showed that DNMTs were recurrently acquired into retrotransposons of dinoflagellates (103). Histone-lysine-*N*-methyltransferases, important in chromatin formation, activation, and transcriptional regulation (104), were identified at ~12-fold higher abundances in the nuclear fraction (shotgun) of *P. cordatum*.

Also, cold shock proteins (CSPs) are widespread in dinoflagellates (105) and the genome of *P. cordatum* contains 59 genes encoding potential CSPs, of which 19 were identified in the present study with ~12-fold higher abundance in the nuclear fraction (geLC). While their specific functions are unclear so far, in *Lingulodinium* and *Symbiodinium,* most CSP-encoding genes are predicted to serve as transcriptional factors (106, 107). By contrast, two dinoflagellate-specific CSPs in *L. polyedra* revealed unspecific nucleic acid-binding properties questioning their role in transcriptional regulation of specific genes (105).

#### Splicing and mRNA processing

The generated protein dataset of *P. cordatum* revealed a high number of small, uridine-rich ribonucleoproteins (sRNPs) of the spliceosome complex (Fig. 6E) (108). All known sRNPs (U1, U2, U4/U6, and U5) could be identified by proteomics in *P. cordatum* as previously described for *L. polyedra* (65). Further assisting proteins of RNA splicing via spliceosome, such as the branch-point binding protein (BBP) and the heterodimeric U2 auxiliary factor (U2AF) (108), could be predicted from the genome of *P. cordatum* (two BBP, one 65 kDa subunit, and three 35 kDa subunit). One BBP and two 35 kDa U2AF subunits could also be identified by proteomics, with higher abundances in the nuclear fractions (geLC, shotgun). Moreover, two components of the exon junction complex, MAGOH (protein mago nashi homolog) and eIF4A III ATP-dependent RNA helicase (eukaryotic initiation factor 4A3) (109), were both identified in *P. cordatum* (~30/50-fold higher abundance of eIF4A III in the nuclear fraction (geLC/shotgun). Taken together, *P. cordatum* forms almost all spliceosome components along with a broad spectrum of other RNA processing proteins (ATP-dependent RNA helicases, initiation and processing factors), cumulatively accounting for approximately one third of the proteins assigned to nuclear functions in the nuclear protein fraction (shotgun). This suggests post-transcriptional regulation to play a major role in this dinoflagellate. Noteworthy, *P. cordatum* possesses multi-codon genes, which are apparently transcribed into a single (polycistronic) transcript, as observed also for other eukaryotes, implicating even post-translational processing (56, 110, 111).

#### Nuclear pore complexes and transport

NPCs are well studied in yeast or human and are built from multiples (~550 and ~1,000 copies, respectively) of ~30 conserved nucleoporins (Nups) (112 - 115). The components of the NPCs in dinoflagellates are largely unknown, and recent identification of NPC components in, e.g., *Toxoplasma gondii* (Apicomplexa) revealed that NPC proteins show, in general, structural conservation across distant eukaryotes (116). The genome of *P. cordatum* encodes only six types of Nups, including Nup49/93 of the inner ring, Nup85 of the cytoplasmic outer ring, Nup1/116 of the nucleoplasmic peripheral structures of the NPC, and one protein with unclear assignment of which only Nup49 and Nup85 could be detected at very low abundances in the present study.

Since the composition of NPCs in *P. cordatum* remains largely unknown at present, this dinoflagellate possibly recruits constituents from the large pool of proteins of unknown function. A BlastP analysis with the functional-studied Nup autopeptidase of *T. gondii* (TgNup302) (116) revealed four possible candidates in *P. cordatum* (e-value: ≤1.0e^−50^). However, all alignments show low coverage (14−24%) and correspondingly rather low sequence identities (~33−40%).

Furthermore, all components of nuclear import and export are encoded in the genome of *P. cordatum*, with RanGTP and an I-subunit also detected on the proteomic level (Fig. 6F). Interestingly, the transport signal on the cargo protein, the so-called nuclear localization sequence (NLS) (117) is currently unknown for dinoflagellates (56). Supplemental background information on the nuclear processes described above in subsections “DNA condensation” to “Nuclear pore complexes and transport” is presented in Fig. S5.

#### Other proteins

In addition to proteins involved in processes, which are exclusively located in the nucleus, further proteins of multi-organizational cellular processes could be identified in the proteomic datasets. These proteins are not only limited to the nucleus and difficult to localize, but could be identified at higher abundances in the nuclear fractions. Their functions comprise ubiquitylation and neddylation, proteasome, biogenesis of translation-competent ribosomal subunits, and initiation of translation. Reconstruction of these processes is illustrated in Fig. S5.

### Conclusion

The FIB/SEM-based 3D reconstruction of the nucleus of *P. cordatum* provided first insights into the number, size range, and packing density of the chromosomes as well as into the spatial distribution of nuclear pores across the nuclear envelope. To further explain the enigmatic structure of the nucleus of *P. cordatum*, growth phase- and environmental condition-dependent dynamics as well as the nuclear net and nuclear pores need to be investigated in greater detail, also taking advantages of further microscopic and spectroscopic approaches, e.g., synchrotron X-ray fluorescence imaging, cryo-electron microscopy (cryo-EM), or nanoscale secondary ion mass spectroscopy.

The here determined proteomic dataset of enriched nuclei of *P. cordatum* establishes a valuable knowledge base for future studies. In particular, the analysis of the recently available genome of *P. cordatum* CCMP 1329 already revealed an extraordinarily high number of proteins of unknown function (62,599; ~73%) (56). In accord, the presented proteomic dataset comprised up to ~37% of such proteins, which are partially highly enriched in the nuclear fractions (Table S3). These proteins may provide promising candidates for nuclear functions and structures, e.g., NPC components, nuclear lamins (118), transcription machinery, and ribosomal assembly. While the genome of *P. cordatum* represents the most complete one of a free-living dinoflagellate, genome analysis revealed distinct gene structures and arrangements (e.g., multi-codon unit genes) challenging functional assignment and hence identification of orthologous (reflected by the BUSCO score of 61.4%) (56). Future proteomic efforts will have to address, among others, the following issues: (i) highly abundant proteins such as chromophore-binding proteins masking low abundant and/or small proteins; (ii) even though the MBNPs were detected at high abundance, that may even be underestimated due to their very small size (~10 kDa); (iii) refine preparation of nuclear envelope and improve extraction of proteins residing therein to increase the chances of detecting further and possibly novel NPC components; (iv) isolate chromosomes with their associated sub-proteome from the enriched nuclei to investigate in more detail the protein components structuring and compacting DNA as well as those possibly contributing to the still elusive process of gene expression.

## MATERIALS AND METHODS

### Cultivation and cell harvest

An axenic culture of *P. cordatum* strain CCMP 1329 was provided by the Helmholtz Centre for Infection Research, Braunschweig, Germany (25). The culture was originally obtained from the Provasoli-Guillard National Center for Marine Algae and Microbiota (formerly the Provasoli-Guillard National Center for Culture of Marine Phytoplankton), Boothbay, ME, USA. The cells were cultivated in synthetic ocean water (SOW) without silicate according to Guillard et al. (119), with slight modification described by Wang et al. (120), at 20°C and under a light intensity of 20 µmol photons m^−2^s^−1^ at a 12-hour:12-hour light-dark cycle. Cells were incubated in a climate chamber (RUMED type P530; Rubarth Apparate, Laatzen, Germany) and were routinely maintained by transferring into fresh medium after 14 days. Sterile controls were conducted at the time points of the cell harvest or inoculation of new cultures by plating aliquots on marine agar (MB) plates. All cultures were cultivated in 120 mL batches in 500-mL Erlenmeyer flasks. The CO_2_ supply was guaranteed by daily shaking. Cell harvest for microscopic and proteomic analyses was conducted consistently after 12 days of cultivation: always 30 minutes after the dark phase of the photoperiodic cycle during the exponential growth. Since mitotic cell division in dinoflagellates mainly occurs in the dark phase, most of the harvested *P. cordatum* cells should be in the interphase (121).

### Light microscopy of cells and nuclei-enriched fractions

#### Light microscopy

For light microscopy, a phase contrast-equipped instrument was used (Primo Star; Carl Zeiss Microscopy GmbH, Oberkochen, Germany) and images were digitalized with an AxioCam ERc 5s camera system (Carl Zeiss Microscopy GmbH). Image processing was performed using the software Zen 2.3 lite (blue edition NT6.1.7601; Carl Zeiss Microscopy GmbH). Differential interference contrast (DIC) microscopy was conducted with a Leica DMRB microscope (Leica Microsystems GmbH, Wetzlar, Germany) at 400× and 640× magnification with oil immersion objectives. Digital micrographs were taken using a Leica DFC420C camera. Image processing was performed using the software Leica Application Suite X.

#### Epifluorescence microscopy

All fractions generated during the nuclei enrichment procedure were inspected by epifluorescence microscopy, using a Zeiss Axioskop 2 (Carl Zeiss Microscopy GmbH) equipped with a 63-fold Zeiss Plan-Neofluar 1.25 oil immersion objective lens and a HB100 mercury lamp for epifluorescence. The excitation wavelength was 365 nm, and the emission was detected at >420 nm (filter set 02: G365, FT395, LP420). Digitalization of images was achieved with an AxioCam 305 color CCD camera (Carl Zeiss Microscopy GmbH) using the ZEN 2.3 SP1 Blue software package (Carl Zeiss Microscopy GmbH). Sample aliquots of 30 µL were incubated with 5 µL DAPI (1 µg/mL stock solution) for at least 15 minutes.

#### Confocal laser scanning microscopy

To document the course of nuclei enrichment, confocal laser scanning microscopy (CLSM) was performed using a Leica TCS SP8 system (Leica Microsystems GmbH). Prior to CLSM, 30 µL concentrated cell material was mixed with 50 µL ProLong Diamond Antifade Mountant with DAPI (Invitrogen, ThermoFisher Scientific, Waltham, MA, USA), embedded on microscope slides (24 × 24, 1.5 mm thickness; Paul Marienfeld GmbH & Co. KG, Lauda-Königshofen, Germany), and dried for 7 days in the dark. Cells were imaged using two optically pumped semiconductor lasers with excitations of 405 nm and 488 nm. Detection was conducted using the Leica hybrid detector for TCS SP8 (HyD) and imaging by means of the Application Suite LAS-X software (version 3.5.7.23225 TCS SP8; Leica Microsystems GmbH).

### Electron microscopy

#### SEM

The specimen preparation for SEM followed the protocol outlined by Nguyen et al. (122), using a culture volume of 50 mL. Cells were fixed with 2.5% glutaraldehyde (vol/vol, diluted in SOW) and incubated at RT for 1 hour. After incubation, the cells were allowed to sediment onto a polyester filter (25 mm in diameter, pore size of 3 µm; Pieper Filter GmbH, Bad Zwischenahn, Germany) and washed four to five times with tap water for 10 minutes each. Specimen dehydration was achieved with a graded ethanol series [30, 50, 70, 80, 90, and 2 × 100% (vol/vol), each for 15–30 minutes]. Following dehydration, the specimen was incubated with hexamethyldisilazan (HMDS) followed by air-drying at RT. For HMDS-mediated drying, the cells on the polyester filters were generally incubated for 15 minutes in 2 mL EtOH:HMDS (1:1, vol/vol) and then for 15 minutes in 1 mL HMDS. Then, the liquid was drained off and the filter air-dried. Afterward, the specimen was coated with 30 nm gold in a sputter coater (SCD 005; BAL-TEC, Walluf, Germany), and examined with a Hitachi S-3200 N SEM operated at 20 kV (Hitachi High Technologies Europe GmbH, Krefeld, Germany). Digitized pictures were taken with the DISS and DIPS software packages (Point Electronic GmbH, Halle, Germany).

#### TEM

The preparation for TEM followed the protocol outlined by Tillmann et al. (123). Cultures (50 mL) were harvested by centrifugation (3,220 g, RT, 10 minutes; Universal 320R; Hettich Zentrifugen, Tuttlingen, Germany) and resuspended in 0.5 mL ice-cold 2.5% glutaraldehyde (in SOW) for 1 hour on ice. Then, the cells were washed two times with 500 µL SOW each and concentrated by centrifugation (16,000 g, 4°C, 10 minutes; Mikro 200R, Hettich Zentrifugen). Postfixation was performed by resuspending the cell pellet in 500 µL of 1% OsO_4_ (vol/vol, diluted in SOW), followed by washing with SOW and concentrating by centrifugation as described above. Dehydration was performed as described above for SEM preparation, with an additional step with 95% ethanol. Further, cells were incubated with propylene oxide in preparation for embedding with EMbed812 resin (Electron Microscopy Science Embed-812 kit; Science Services GmbH, München, Germany). Samples were finally transferred into BEEM capsules (Serva, Heidelberg, Germany) and polymerized at 60°C overnight.

After ultrathin sectioning, the samples were investigated using a Zeiss EM912 (Carl Zeiss Microscopy GmbH), operated at 80 kV, and equipped with a Tröndle 2k × 2k slow-scan CCD camera (TRS, Tröndle Restlichtverstärkersysteme, Moorenweis, Germany) or a JEOL F200 (JEOL Germany, Freising, Germany), operated at 200 kV and equipped with a EMSIS 20-megapixel CMOS camera (EMSIS GmbH, Münster, Germany).

#### FIB/SEM

Samples for FIB/SEM series (FIB/SEM tomography) were pretreated, fixed, and embedded as described for TEM. After polymerization for 96 hours at 60°C, the trimmed resin block was mounted on a thin aluminum pin and coated with 10 nm carbon. Milling and imaging of the samples were carried out with an Auriga 40 FIB/SEM workstation using the SmartSEM software package (Carl Zeiss Microscopy GmbH). Images were recorded with an acceleration voltage of 1.5 kV by using an EsB detector with the EsB-grid set to 500 V and the 30 µm aperture. The scan speed was set to an exposure time of 90 seconds for each image with a total size of 2,048 × 1,536 pixels. For milling, an ion beam current of 100 pA with a milling rate that resulted in 20 nm slices was used. Images were then always taken in multitudes of these 20 nm. The voxel sizes depended on the investigated structure and ranged between 10 and 15 nm in x/y and 20 or 30 nm in z for whole cells. When focusing on the nucleus to display nuclear pores (Fig. 2A), we used iso-voxels of 12 × 12 × 20 nm in x/y/z direction.

### 3D reconstruction of the nucleus

The 3D reconstruction of the nucleus of *P. cordatum* was performed using the multifaceted image analysis software Amira (Amira2020.3.1; ThermoFisher Scientific). Prior to 3D visualization, the 265 TIF images were transformed to one TIF data file by the open-source image processing platform FIJI (ImageJ 1.53e) (124). In Amira, the structures of the nucleus were drawn as material in the segmentation editor with the help of an external creative pen display (Wacom Cintiq 22; Wacom K.K., Kazo, Saitama, Japan). The visualization of the materials was conducted using the application tools *Generate surface* and *Surface view*. All structures were subjected to smoothing by using the property *Unconstrained smoothing* with a smoothing extent of 9. The unknown structure was smoothed with a smoothing extent of 5. For displaying the structures in their respective *Surface view*, the drawn style for each material was set to shaded, except for the nuclear membrane. The application of this structure was set to transparent with a base transparency of 0.7. Volume calculations of the individual structures were conducted using the application tool *Material statistics*. The generated volumes in Voxel were further recalculated to volume µm^3^. To illustrate the nuclear membrane with nuclear pores, the *Surface view* was set to shaded.

### Nuclei enrichment

The enrichment of nuclei of *P. cordatum* followed the protocol given below and is summarized in Fig. 2. Cells from the exponential growth phase were harvested by centrifugation (1,000 g, RT, 5 minutes; Eppendorf 5920R; Eppendorf AG, Hamburg, Germany) and washed once with SOW to remove culture remains. Prior to cell disruption, the cells were resuspended in nuclei isolation buffer (NIB: 250 mM sucrose, 50 mM Tris/HCl, pH 7.4, 1 mM EDTA) containing 30% ethanol adapted from Levi-Setti et al. (68), washed two times with NIB, and finally resuspended in 1 mL NIB. For cell disruption, the samples were sonicated for 1 minute (Branson Ultrasonics Sonifier 250 CE; ThermoFisher Scientific) with an output of 30% and a duty cycle of 25% on ice. Then, the sample was subjected to a first fractionation by a three-layer discontinuous Percoll-sucrose gradient (70,000 g, 4°C, 90 minutes; Beckmann Avanti J 25.5; Beckmann Coulter, Krefeld, Germany) according to Schikowsky et al. (69). Further enrichment and purification of nuclei was achieved by a five-layer discontinuous Percoll-sucrose gradient. In order to remove larger contaminants, the final fraction was washed three times with a modified NIB wash buffer [125 mM sucrose, 35 mM Tris/HCl, pH 7.4, 0.5 mM EDTA, 2.5 mM MgCl_2_, and 0.5% TritonX100 (vol/vol)] and centrifuged at 1,000 g and 4°C for 10 minutes (Eppendorf 5920 R; Eppendorf AG). The final nuclei pellet was shock frozen in liquid nitrogen and stored at –80°C until usage. Integrity along the sample preparation was controlled by epifluorescence light microscopy (see above).

### Preparation of (sub-)cellular protein fractions

For solubilization of the total nuclear protein (TNP), nuclei pellets were resuspended in 1% (wt/vol) SDS and cells disrupted by means of bead beating (Fast-Prep-24 5G; MP Biomedical, Eschwege, Germany) for 10 seconds at 6.5 m s^−1^ followed by 90 seconds on ice (three repetitions) using 1 mm silica beads. Protein solubilization was facilitated by incubation for 10 minutes at 95°C (Thermomixer comfort; Eppendorf AG) and nuclei debris removed by centrifugation (20,817 g, 20°C, 10 minutes; Eppendorf 5427 R; Eppendorf AG). The final protein was shock frozen in liquid nitrogen and stored at –80°C. In addition to the TNP fraction, the following two subnuclear fractions were prepared: nuclear soluble (NSP) and nuclear membrane (NMP) fractions. For NSP, nuclear proteins were extracted with shotgun-lysis buffer (SG-LB; 7 M urea, 2 M thiourea, 30 mM Tris/HCl, pH 8.0), nuclei disrupted as described above, and nuclei fragments removed by centrifugation at 104,000 g and 10°C for 60 minutes (Beckmann Coulter). The supernatant was shock frozen in liquid nitrogen and stored at –80°C. In case of NMP, the nuclei pellet was resuspended in ice-cold membrane lysis buffer [10% glycerol (vol/vol), 30 mM Tris/HCl, pH 8.0, 5 mM MgCl_2_] and nuclei disrupted as described above. Subsequently, the membrane fraction was washed twice with ice-cold membrane lysis buffer and centrifuged at 104,000 g and 4°C for 60 minutes. Proteins were solubilized with 1% (wt/vol) SDS, facilitated by incubation at 600 rpm and 95°C for 10 minutes. Nuclei fragments were removed by centrifugation as described above. As reference, the same procedures as for NSP and NMP, respectively, were applied to whole cell preparations, yielding CSP and CMP fractions. All protein fractions were prepared in triplicate. The protein concentration of the soluble protein fractions (NSP and CSP) were determined according to the Bradford method using a commercial assay (Bio-Rad Laboratories, Munich, Germany). For proteins extracted with SDS (NTP, NMP, and CMP) the detergent compatible RC DC assay was applied (Bio-Rad Laboratories), using BSA as standard in both cases (Sigma-Aldrich Inc., Hamburg, Germany).

### Protein separation

To achieve high resolution, each triplicate of the five protein fractions was separated by continuous gradient (5.5–14% acrylamide) 1D SDS PAGE with a separation distance of 20 cm and a gel thickness of 1 mm (125). A total of 30 µg protein per sample was applied and 5 µL marker (Serva triple color protein standard III; Serva Electrophoresis GmbH, Heidelberg, Germany) was loaded. Post-electrophoretic staining of the proteins was performed using silver as described by Yan et al. (126).

### Mass spectrometric analysis and protein identification

For mass spectrometric protein identification, the entire lane of a given gel-separated fraction was cut into 34 gel slices. Each slice was further cut into small pieces of ~1–2 mm^3^ for subsequent in-gel digest as described (127). Extracted peptides were subjected to nanoLC separation (Ultimate 3000 nanoRSLC; ThermoFisher Scientific) using a trap column setup (2 cm length, 5 µm bead size, 75 µm inner diameter; ThermoFisher Scientific) and applying a 90-minute linear gradient. The eluent was continuously ionized (captive spray ion source; Bruker Daltonik GmbH, Bremen, Germany), and ions were analyzed by an ion-trap mass spectrometer (amaZon speed ETD; Bruker Daltonik GmbH) as described (127). Protein identification was performed using Mascot (version 2.3; Matrix Science, London, UK) via the ProteinScape platform (version 4.2; Bruker Daltonik GmbH) and a genomic database of *P. cordatum* (56). A target-decoy strategy with a false discovery rate <1.0% was applied as well as the following settings: enzyme trypsin; one missed cleavage allowed, carbamidomethlyation (C) as fixed, oxidation (M) as variable modification; peptide and MS/MS mass tolerance 0.3 Da; monoisotopic; peptide charge 2+ and 3+; instrument type ESI-TRAP; significance threshold *P* < 0.05; ion score cutoff 25.0; minimum peptide length of 5. The search results of all 34 slices per sample were compiled using the ProteinExtractor function.

Sample preparation for label-free liquid chromatography coupled tandem mass spectrometry (LF-LC-MS/MS) was based on the SP3 protocol outlined in Mikulášek et al. (128), with minor adjustments. For peptide clean-up and desalting, dried samples were taken up in 100 µL of 1% formic acid and loaded onto SepPak C18 1 cc columns (Waters, Eschborn, Germany), mounted onto a vacuum-operated extraction manifold (fitting 20 cartridges). Wetting, washing, equilibration, sample loading, and peptide elution were performed according to the manufacturer’s instructions. Dried peptides were resuspended in 100 µL of 0.1% formic acid, and peptide concentrations were tested using a colorimetric, bicinchoninic acid-based assay (ThermoFisher Scientific) according to the manufacturer’s instructions. A volume corresponding to 2 µg of peptides was transferred to new low-binding reaction tubes and dried in a vacuum centrifuge, before being resuspended in 10 µL of formic acid, resulting in a final peptide concentration of 200 ng/µL.

LF shotgun mass spectrometry (MS) was performed using a timsTOF Pro instrument coupled to a nanoElute UPLC (both Bruker Daltonik GmbH). Peptides were injected using the manufacturer’s standard procedure and were subsequently separated on a 25-cm Aurora nanoZero column equipped with a Captive Spray Insert (IonOpticks, Fitzroy, Australia) using a 60-minute acetonitrile gradient ranging from 2% to 37% acetonitrile in 0.1% formic acid. Column temperature was set to 50°C and a flow rate of 400 nL min^−1^ was employed.

MS2 spectra were obtained in positive mode using the DDA PASEF standard preinstalled in the parallel accumulation-serial fragmentation (PASEF) workflow. Protein identification and quantitation was achieved using the MaxQuant software package [v2.0.2.0 (129)]. For identification of proteins, standard settings were selected. MS/MS spectra were queried against an in-house *P. cordatum* protein database.

Since the two approaches generate different types of protein abundance information (peptide counts, geLC; IBAQ, shotgun; see Materials and Methods for details), calculated relative shares per protein and fraction were used for comparison of the two datasets.

### Functional assignment and bioinformatic protein localization prediction

Protein sequences of *P. cordatum* were subjected to localization prediction using WoLF PSORT (130) and DeepLoc (131). In contrast to other predictors, DeepLoc applies deep neural networks for subcellular localization prediction (131). In case of proteins with (multiple) isoforms, the prediction for the experimentally detected protein was used. Besides subcellular localization prediction, sequences of characterized nuclear proteins from the NLSdb (132) were used for BLAST search against the proteins of *P. cordatum*. In addition to localization predictions, ontology-based allocation to KEGG pathways was used to predict proteins with nuclear function (56, 133), considering the following KEGG pathway maps: genetic information processing (transcription, translation, folding and degradation, replication, and repair) and cell growth and death (cell cycle).

## Data Availability

The proteomic data have been deposited at FAIRDOMHub (https://‌fairdo‌m‌hub.‌org/‌projects/‌300). The movies 1 and 2 have been deposited at FAIRDOMHub (https://‌fairdomhub.‌org/‌data_files/‌6144?‌code=‌L4cddC0wR0VQzKkfh%‌2FCBryGfiRKpuMsVGNzbXKdq and https://‌fairdomhub.‌org/‌data_files/‌6145?code=‌eDGAaJGquOSwDy795LdYq5BzZ1DJ0sQbsQepZf8J).

## SUPPLEMENTAL MATERIAL

Fig S1 msphere.00038-23-s0001.pdf Morphology of *P. cordatum.*

Fig S2 msphere.00038-23-s0002.pdf 2D-FIB-/SEM images and 3D reconstruction of the conspicuous structure in the nucleus of *P. cordatum.*

Fig S3 msphere.00038-23-s0003.pdf Image plate of the chromosomes with their respective volumes in *P. cordatum*.

Fig S4 msphere.00038-23-s0004.pdf Supplementary proteomic data of *P. cordatum.*

Fig S5 msphere.00038-23-s0005.pdf Nuclear and multi-organizational cellular processes.

TABLE S1 msphere.00038-23-s0006.pdf Literature data for comparison of *P. cordatum* genome size and chromosome numbers.

TABLE S2 msphere.00038-23-s0007.pdf Tested protocols for nuclei enrichment of *P. cordatum*.

TABLE S3 msphere.00038-23-s0008.pdf Top ten of unknown proteins of *P. cordatum*.

TABLE S4 msphere.00038-23-s0009.pdf Sequence-based identification of ribosomal subunits detected for *P. cordatum*.

ASM does not own the copyrights to Supplemental Material that may be linked to, or accessed through, an article. The authors have granted ASM a non-exclusive, world-wide license to publish the Supplemental Material files. Please contact the corresponding author directly for reuse.
