# Supplementary material for: The enigmatic nucleus of the marine dinoflagellate Prorocentrum cordatum
Source: mSphere. 2023 Jun 26;8(4):e00038-23. doi: 10.1128/msphere.00038-23 (PMC10449503; doi:10.1128/msphere.00038-23)
Supplement: Fig S1 — Morphology of P. cordatum. [file msphere.00038-23-s0001.pdf]

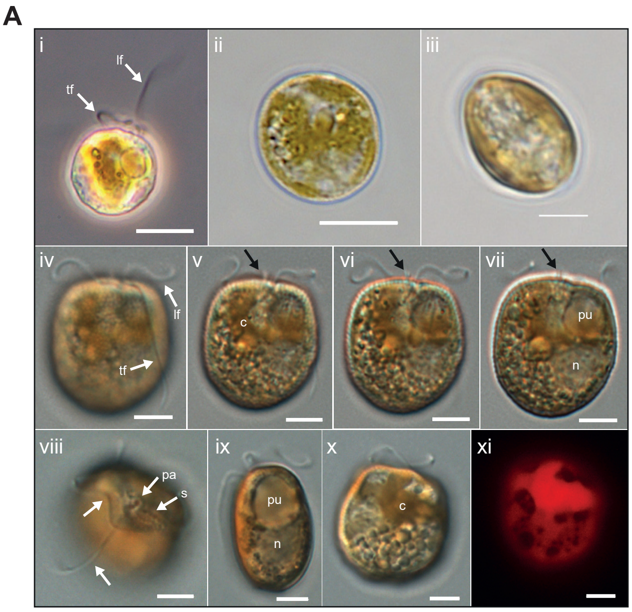

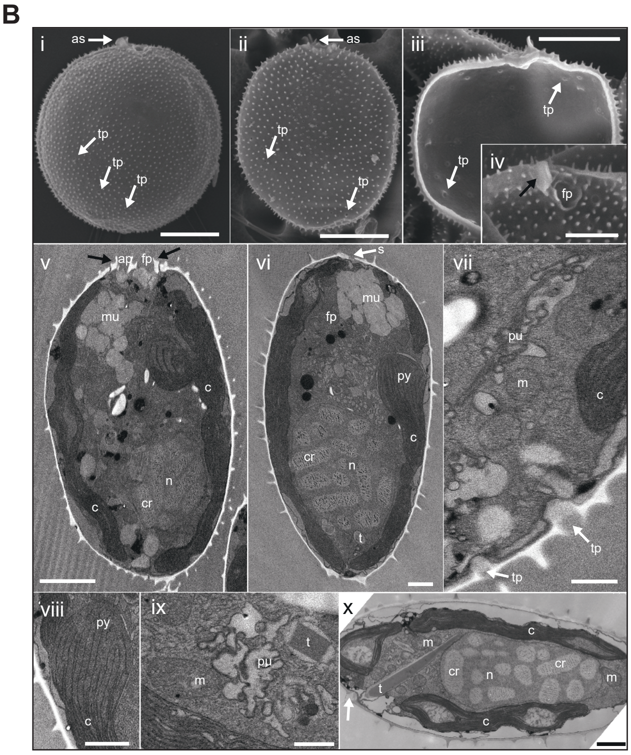

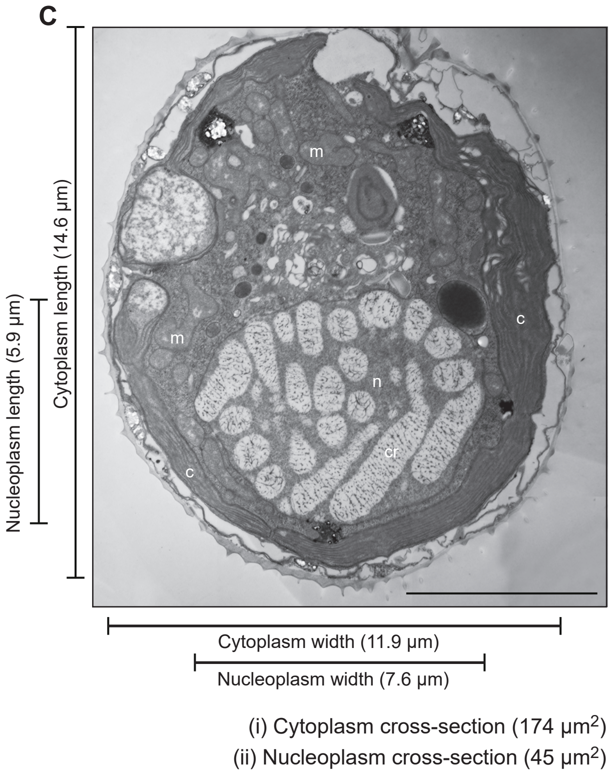

Fig. S1. Morphology of *P. cordatum*. (**A**) Light microscopic (LM) images. (**i-iii**) Phase contrast, (**iv-x**) differential interference contrast, and **(xi)** epifluorescence microscopic images. (**i**) Cell in lateral view showing the apical insertion of the two flagella (desmokont flagellation). Note that the longitudinal flagellum (lf) is directed apically and the transverse flagellum (tf) beats outside any furrow around the anterior cell end. (**ii**) Cell in lateral view. (**iii**) Cell in sagittal suture view (likely dorsal or ventral) in about mid cell focus showing the flattened cell shape and the chloroplasts located peripheral below the lateral thecal plates. (**iv-vii**) Same cell in lateral view in different focal planes. (**iv**) Surface focus showing the spiny thecal ornamentation and the two flagella, transverse (tf) and longitudinal (lf). (**v-vi**) Apical spine-like double-structure (black arrows); chloroplast (c), anterior pusule (pu) and part of the nucleus (n). (**viii**) Apical view with focus on the small periflagellar area (pa). Note the sagittal suture (s) and the two flagella. (**ix**) Ventral view demonstrating the flattened cell shape. Focus through the pusule (pu) and nucleus (n). (**x**, **xi**) Same cell showing part of one lobed chloroplast (c). Scale bars: **i** = 10 µm; **ii-xi** = 5 µm. (**B**) Electron microscopic (EM) images of *P. cordatum*. (**i-iv**) Scanning EM (SEM). (**v-x**) Transmission EM (TEM). (**i**, **ii**) Right lateral view of the cell showing the spiny thecal ornamentation and thecal pores (tp) and the small apical indentation of the periflagellar area with the tiny apical wing-like spines on the dorsal platelet 1 (as). (**iii**) Right thecal plate with periflagellar platelets in inside view more clearly showing the large thecal pores (arrows). (**iv**) Periflagellar area with apical wing-like spines (black arrow) and flagellar pore (fp). (**v**) Longitudinal section in dorsoventral direction displaying parts of the periflagellar platelets (black arrows), the accessory pore (ap) and the flagellar pore (fp). (**vi**) Longitudinal section in lateral direction with sagittal suture between the two large lateral thecal plates (s). (**vii**) Detail showing part of the pusule (pu) and thecal pores (tp). (**viii**) Detail of the pyrenoid (py) as part of the chloroplast (c). (**ix**) Detail showing a mitochondrium (m), pusule (pu) and a transverse section of a trichocysts (t). (**x**) Transverse section in the lower cell half with longitudinal section of a trichocyst (t). Scale bar: **i-iii** = 5 µm; **iv** = 2 µm; **v** = 2.5 µm; **vi**, **viii**, **x** = 1 µm; **vii**, **ix** = 0.5 µm. (**C**) TEM of cell ultrastructure of *P. cordatum*. Abbreviations: c, chloroplast; cr, chromosome; m, mitochondrium; n, nucleus; Scale bar: 5 µm.

Morphology of *P. cordatum*

Phase contrast, differential interference contrast (DIC) and epifluorescence microscopy revealed the desmokont flagellation (Fig. S1Ai) and the general cell shape, round to oval in lateral (Fig. S1Aii) and laterally flattened lense-shaped in sagittal suture view (Fig. S1Aiii). The yellowish/brownish chloroplasts (c) are easily recognizable (Figs. S1Ai-x) by light microscopy and can be verified by their autofluorescence with epifluorescence microscopy (Fig. S1Axi). The tiny apical spine-like double-structure (black arrows), pusule (pu), nucleus (n) and the spiny thecal surface are visible already with DIC in the light microscope with high magnification (Figs. S1Aiv-x). Scanning electron microscopy (SEM) showed the thecal plates densely covered with spines and perforated by thecal (likely trichocyst) pores (tp) (Figs. S1Bi-iii) (1-3). The sagittal suture (s) was transversely striated (not shown). Unfortunately, the microarchitecture of the periflagellar area (pa) was not clearly visible in our preparations, but the apical wing-like spines (as) on platelet 1 were visible (Figs. S1Bi-iv) (4). Transmission electron microscopy (TEM) allowed to visualize subcellular compartmentation (Figs. S1Bv-vi), in particular the marked space requirement of the nucleus (approx. ¼ of cytoplasm in cross-section, Fig. S1C) and chromosomes therein (Figs. S1Bv,v,ix). A longitudinal section in lateral direction (Fig. S1Bv) and a transverse section in the lower cell half (Fig. S1Bx) revealed the flattened cell shape, which the nucleus (n) mirrors. A longitudinal section in dorsoventral direction showed parts of the periflagellar platelets (black arrows), the accessory pore (ap) and the flagellar pore (fp) (Fig. S1Bv). Mucocysts (mu) in the apical cell part below the periflagellar area, trichocysts (t), the peripherally arranged chloroplasts (c), the pusule (pu), and typical mitochondria (m) with tubular cristae were visible (Figs. S1Bv-x). A terminal pyrenoid (py) with intruding thylakoids was recognized (Figs. S1Bvi,viii). Since these microscopic analyses are well in accord with previous reports of other *Prorocentrum* species (5, 6) our present analysis of the nucleus of *P. cordatum* should represent a fitting expansion of the knowledge on the subcellular structure of this genus.

References

1. Hoppenrath M, Elbrächter M, Drebes G. 2009. Marine phytoplankton. Selected microphytoplankton species from the North Sea around Helgoland and Sylt. *In* Kleine Senckenberg-Reihe 49:1-264.
2. Pertola S, Faust MA, Kuosa H, Hällfors G. 2003. Morphology of *Prorocentrum minimum* (Dinophyceae) in the Baltic Sea and in Chesapeake Bay: Comparison of cell shapes and thecal ornamentation. Botanica Marina 46:477-486.
3. Faust M, Larsen J, Moestrup Ø. 1999. Potentially toxic phytoplankton. 3. *Prorocentrum* (Dinophyceae) ICES 184:1-24.
4. Monti M, Stoecker DK, Cataletto B, Talarico L. 2010. Morphology of the flagellar pore complex in *Prorocentrum minimum* (Dinophyceae) from the Adriatic and Baltic Seas. Botanica Marina 53:357-365.
5. Hoppenrath M, Chomérat N, Horiguchi T, Schweikert M, Nagahama Y, Murray SA. 2013. Taxonomy and phylogeny of the benthic *Prorocentrum* species (Dinophyceae) - A proposal and review. Harmful Algae 27:1-28.
6. Hoppenrath M, Leander BS. 2008. Morphology and molecular phylogeny of a new marine sand-dwelling *Prorocentrum* species, *P. Tsawwassenense* (Dinophyceae, Prorocentrales), from Britisch Columbia, Canada. J Phycol 44:451-466.
