# Supplementary material for: The enigmatic nucleus of the marine dinoflagellate Prorocentrum cordatum
Source: mSphere. 2023 Jun 26;8(4):e00038-23. doi: 10.1128/msphere.00038-23 (PMC10449503; doi:10.1128/msphere.00038-23)
Supplement: Fig S2 — 2D-FIB-/SEM images and 3D reconstruction of the conspicuous structure in the nucleus of P. cordatum. [file msphere.00038-23-s0002.pdf]

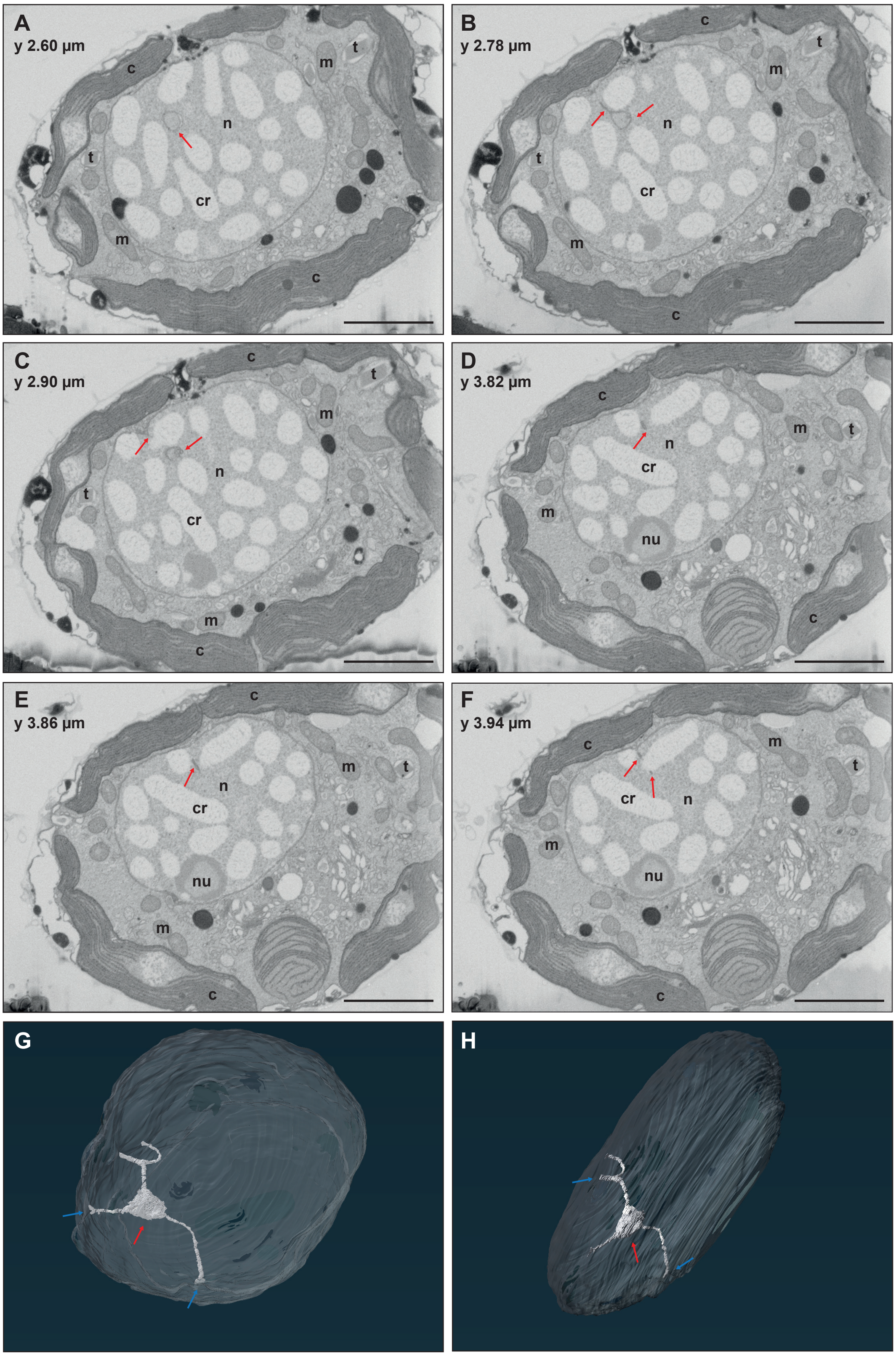

Fig. S2. 2D-FIB-/SEM images and 3D reconstruction of the conspicuous structure in the nucleus of *P. cordatum.* (**A**-**H**) Conspicuous structure in 2D- and 3D-view (red arrows). (**G**, **H**) Bonding of the conspicuous structure with the nuclear membrane (blue arrows) in 3D-view. Scale bar: **A**-**F** = 5 µm, depth (y): **A**-**F** = 20 nm. Abbreviations: c, chloroplast; cr, chromosome; m, mitochondrium; n, nucleus; nu, nucleolus; t, trichocyst.
