# Supplementary material for: The enigmatic nucleus of the marine dinoflagellate Prorocentrum cordatum
Source: mSphere. 2023 Jun 26;8(4):e00038-23. doi: 10.1128/msphere.00038-23 (PMC10449503; doi:10.1128/msphere.00038-23)
Supplement: Fig S3 — Image plate of the chromosomes with their respective volumes in P. cordatum. [file msphere.00038-23-s0003.pdf]

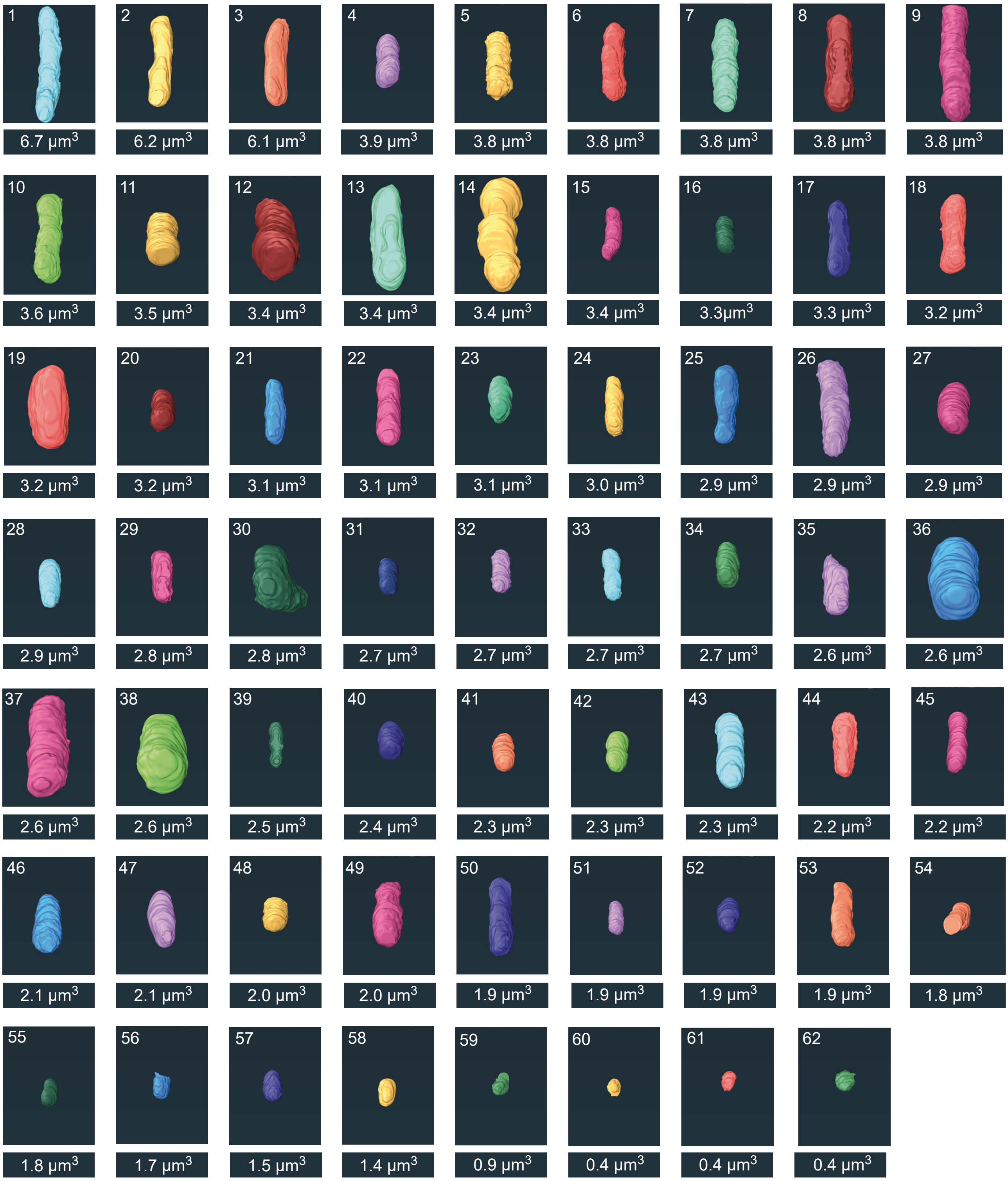

Fig. S3. Image plate of the chromosomes with their respective volumes in *P. cordatum*. Further details are provided in: Pcordatum_FigS3_3D reconstruction volume calculation.xlsx.
