# Supplementary material for: The enigmatic nucleus of the marine dinoflagellate Prorocentrum cordatum
Source: mSphere. 2023 Jun 26;8(4):e00038-23. doi: 10.1128/msphere.00038-23 (PMC10449503; doi:10.1128/msphere.00038-23)
Supplement: Fig S4 — Supplementary proteomic data of P. cordatum. [file msphere.00038-23-s0004.pdf]

A

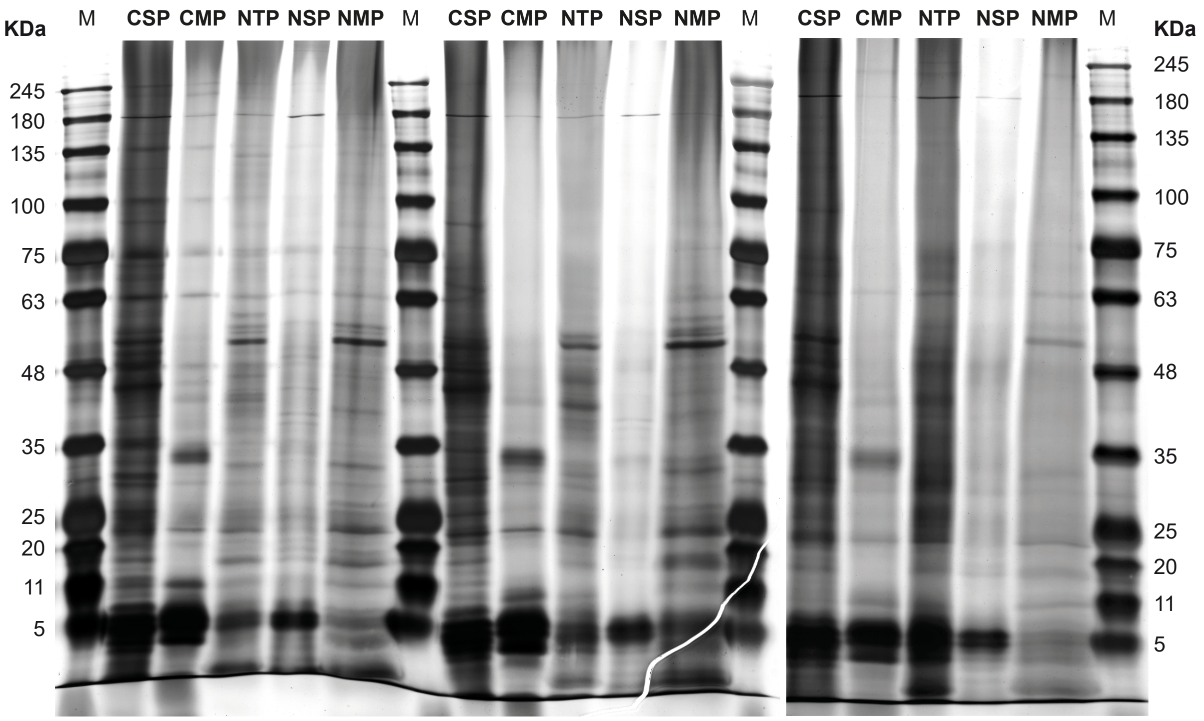
KDa
M
CSP
CMP
NTP
NSP
NMP
M
CSP
CMP
NTP
NSP
NMP
M
CSP
CMP
NTP
NSP
NMP
M
KDa
245
180
135
100
75
63
48
35
25
20
11
5
245
180
135
100
75
63
48
35
25
20
11
5

B

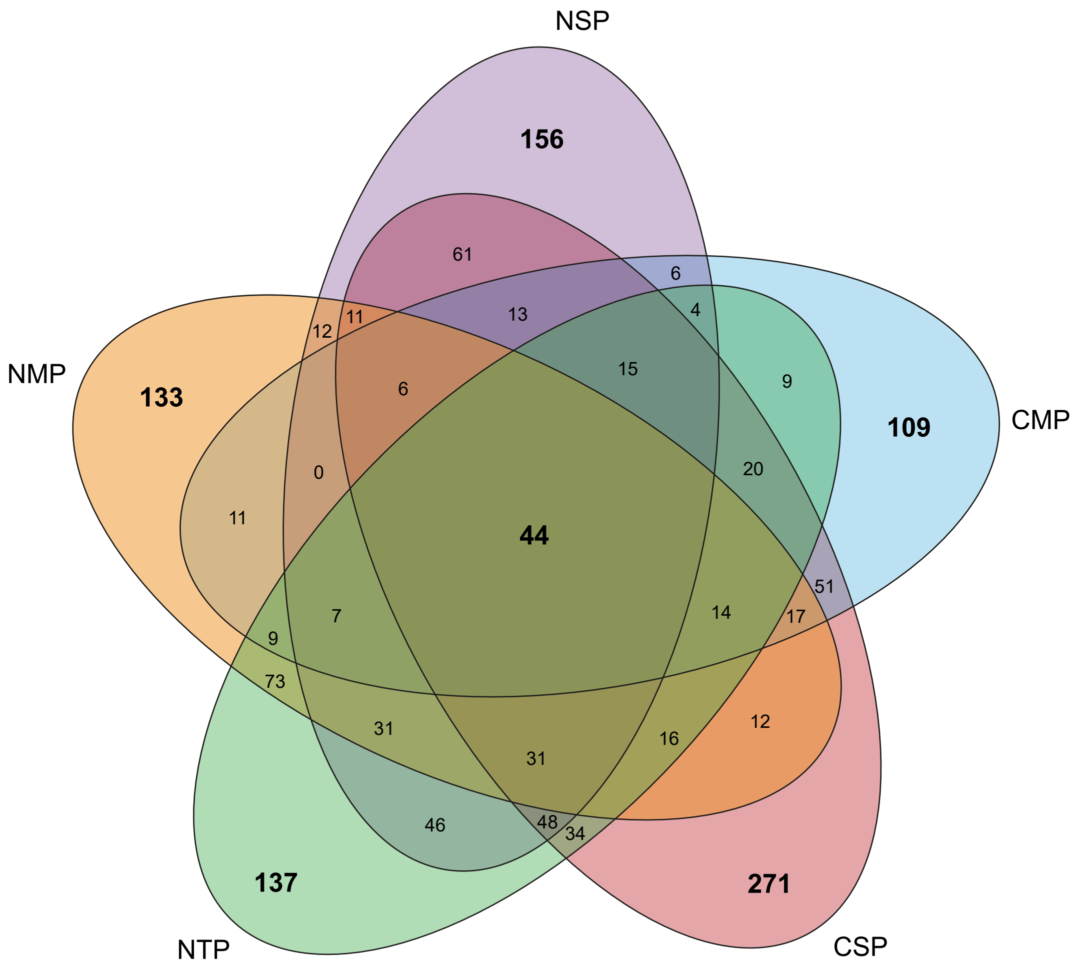
NSP
156
NMP
133
CMP
109
NTP
137
CSP
271
44
61
6
13
4
12
11
15
9
6
20
0
11
51
7
14
17
9
73
31
16
12
31
46
48
34

C

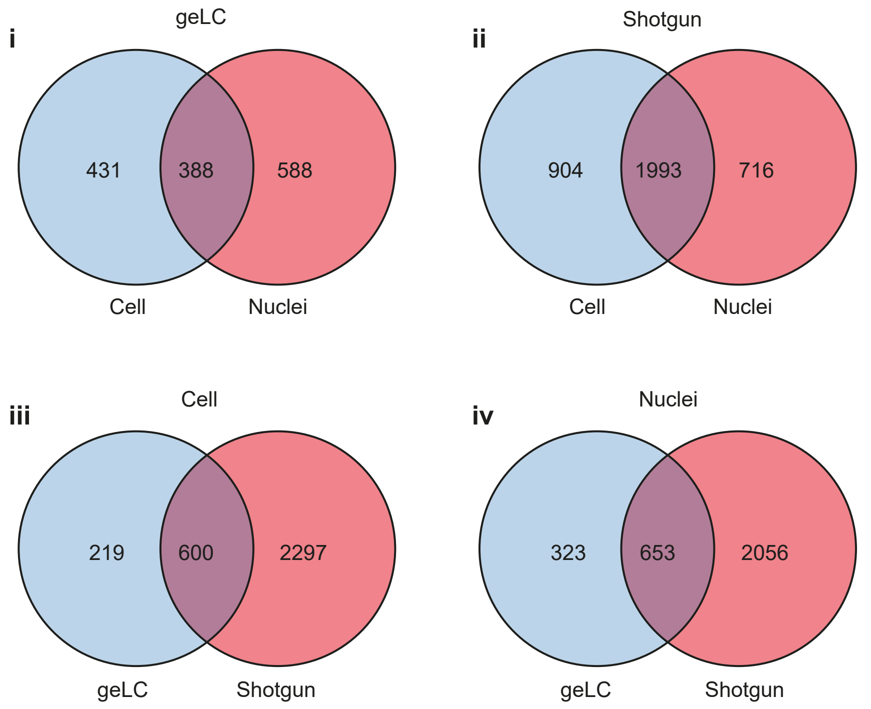
i
geLC
431
388
588
Cell
Nuclei
ii
Shotgun
904
1993
716
Cell
Nuclei
iii
Cell
219
600
2297
geLC
Shotgun
iv
Nuclei
323
653
2056
geLC
Shotgun

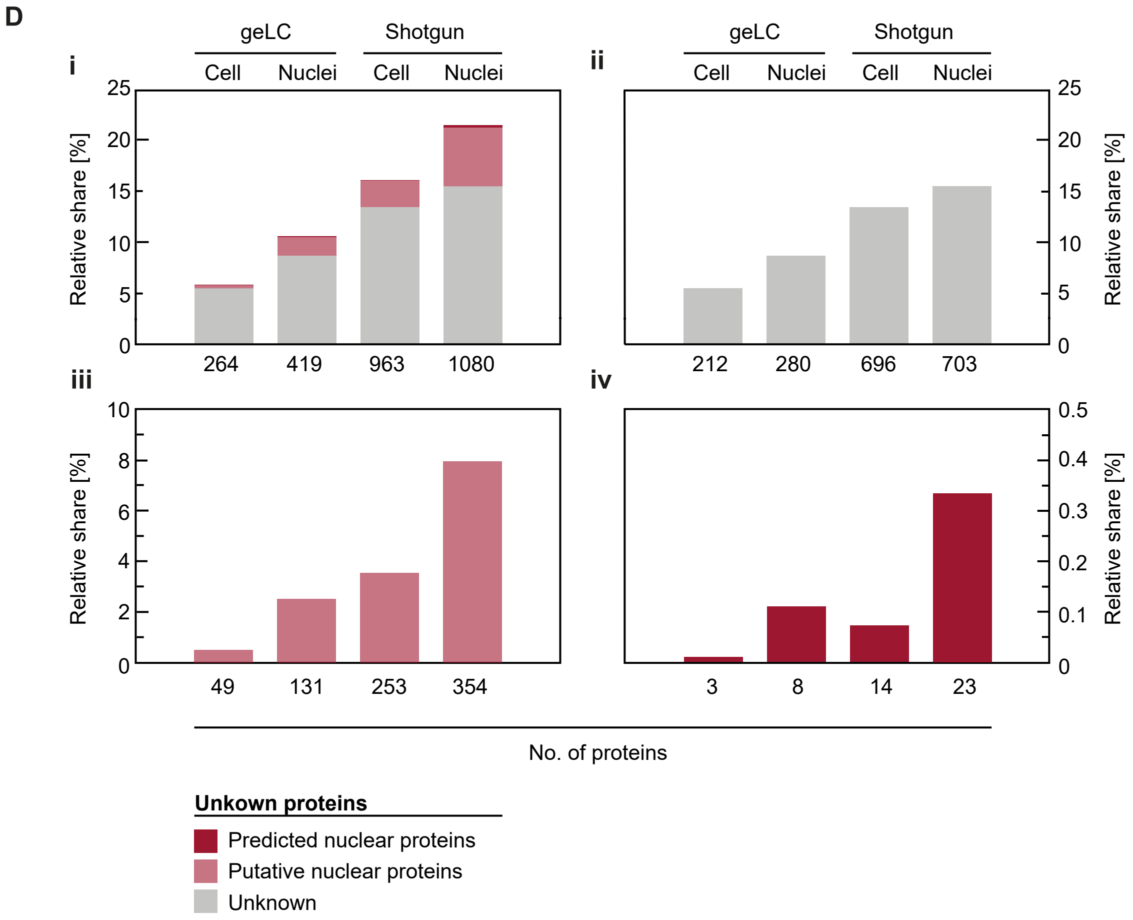

Fig. S4. Supplementary proteomic data of *P. cordatum*. (**A**) Separation of prepared subcellular fractions of *P. cordatum* via gradient SDS-PAGE (silver-stained), replicate R1–R3. (B) Shared number of identified proteins for P. cordatum for the 5 different protein fractions.(**C**) Number of identified proteins for *P. cordatum*. (**i**) Individual and shared number of proteins of the geLC approach. (**ii**) Individual and shared number of proteins of the shotgun approach. (**iii**) Individual and shared number of proteins of the cellular fraction. (**iv**) Individual and shared number of proteins of the nuclear fraction. (**D**) Relative share of proteins and numbers of unknown proteins and putative/predicted nuclear proteins of *P. cordatum*. (**i**) Summary. (**ii**) Unknown proteins. (**iii**) Putative nuclear proteins. (**iv**) Predicted nuclear proteins. Further details are provided in: Pcordatum_Proteomic_Tab1_data summary.xlsx and Pcordatum_Proteomic_Tab3_prediction.xlsx
