# Supplementary material for: The enigmatic nucleus of the marine dinoflagellate Prorocentrum cordatum
Source: mSphere. 2023 Jun 26;8(4):e00038-23. doi: 10.1128/msphere.00038-23 (PMC10449503; doi:10.1128/msphere.00038-23)
Supplement: Fig S5 — Nuclear and multi-organizational cellular processes. [file msphere.00038-23-s0005.pdf]

Further background on nuclear processes

In the following, a short introduction into the different nuclear processes is given to guide the readers through the most important functions of nuclear proteins.

(i) DNA condensation. In almost all eukaryotic cells, condensation of nuclear DNA starts by wrapping the DNA double helix around core histones, forming nucleosomes which are further twisted via chromatin fibers to chromatin loops and compartments finally yielding the compact chromosomes (for overview see (1)). Furthermore, histone chaperone networks play multiple roles in chromatin functioning (2), spatio-temporal dynamics of nuclear compartmentalization and gene expression control (3).

(ii) DNA replication. DNA replication is a crucial process for every cell to divide, executed by an extraordinarily accurate protein machinery and usually occurring at the de condensed chromosomes during the interphase of mitosis/meiosis (4, 5).

(iii) RNA processing. The nuclear exosome complex is a multifunctional RNA quality control system in eukaryotes, responsible for RNA maturation, surveillance and degradation and might be further involved in regulation of mRNA expression (6). The individual RNA molecule is recognized by exosome-specificity factors (ESFs) to initiate degradation. To date, only a few ESFs are known for eukaryotes, as e.g. the nucleolar RNA-processing factor 53 in *S. cerevisiae.* After recognition, the RNA substrate is activated for degradation by binding of accessory proteins and RNA helicase (6). Finally, the RNA is degraded within the nuclear exosome complex (6-8).

(iv) Transcription. In eukaryotes, a six-multi subunit complex consisting of interacting transcription factors (TFs) binds at a specific core promoter sequence (TATA-box/GC-box) to initiate transcription, performed by RNA polymerase II (RNAP2) (9-13). The protein, which usually binds to the canonical promoter, is the TATA-box-binding protein (TBP), a subunit of the eukaryotic transcriptional factor TFIID (14).

(v) Splicing and mRNA processing. Newly synthesized precursor mRNA (pre-mRNA) is processed in the nucleus prior to export into the cytoplasm where it serves as template for protein biosynthesis (18, 19). This processing involves pre-mRNA splicing, where non-coding introns (intervening sequences) are removed from the pre-mRNA to form mature mRNA (20). This splicing is performed by the spliceosome, which is mainly composed of several small, uridine-rich ribonucleoproteins (sRNPs) (18).

(vii) Nuclear pore complexes and transport. The selective import and export of macromolecules (RNA molecules, proteins or ribosomal subunits) across the nuclear envelope (NE) is mediated by large (~50–120 MDa) NE-embedded nuclear pore complexes (NPCs) (21-24). NCPs are well studied in yeast or human and are built from multiples (~550 and ~1,000 copies, respectively) of ~30 conserved nucleoporins (Nups) forming an inner scaffold overlayed by peripheral structures (21-24). The scaffold consists of an inner ring sandwiched between a cytoplasm-facing outer ring and a nucleoplasm-facing membrane ring, giving rise to a ~500 Å wide central channel (21-24). The NPCs are completed towards the cytoplasm by the export platform and towards the nucleoplasm by the nuclear basket (21-24).

Cytoplasmic cargo designated for nuclear import (Fig 6F, left panel) carries a so-called nuclear localization sequence (NLS), which is rich in basic residues (25); NLS in dinoflagellates is currently unknown (26). The NLS is recognized by importin α (Iα), which interacts with importin β (Iβ) (25). Upon interaction of the latter with the NPC, translocation is initiated. In the nucleoplasm the importins dissociate from the cargo and are translocated back into cytoplasm as Iβ/RanGTP and Iα/CAS/RanGTP complexes through the NPCs (25). In case of Iα NUP50 is involved in nucleoplasmic Iα/CAS/RanGTP complex formation and GAP in cytoplasmic Iα liberation from this complex (27).

Nucleoplasmic cargo designated for nuclear export (Fig 6F, right panel) carries a so-called nuclear export sequence (NES) (28). Upon formation of an exportin(E)-cargo-RanGTP complex in the nucleoplasm translocation across the NPC occurs (28). In the cytoplasm, disassembly of the E-cargo-RanGTP complex yields directionality of the export (28).

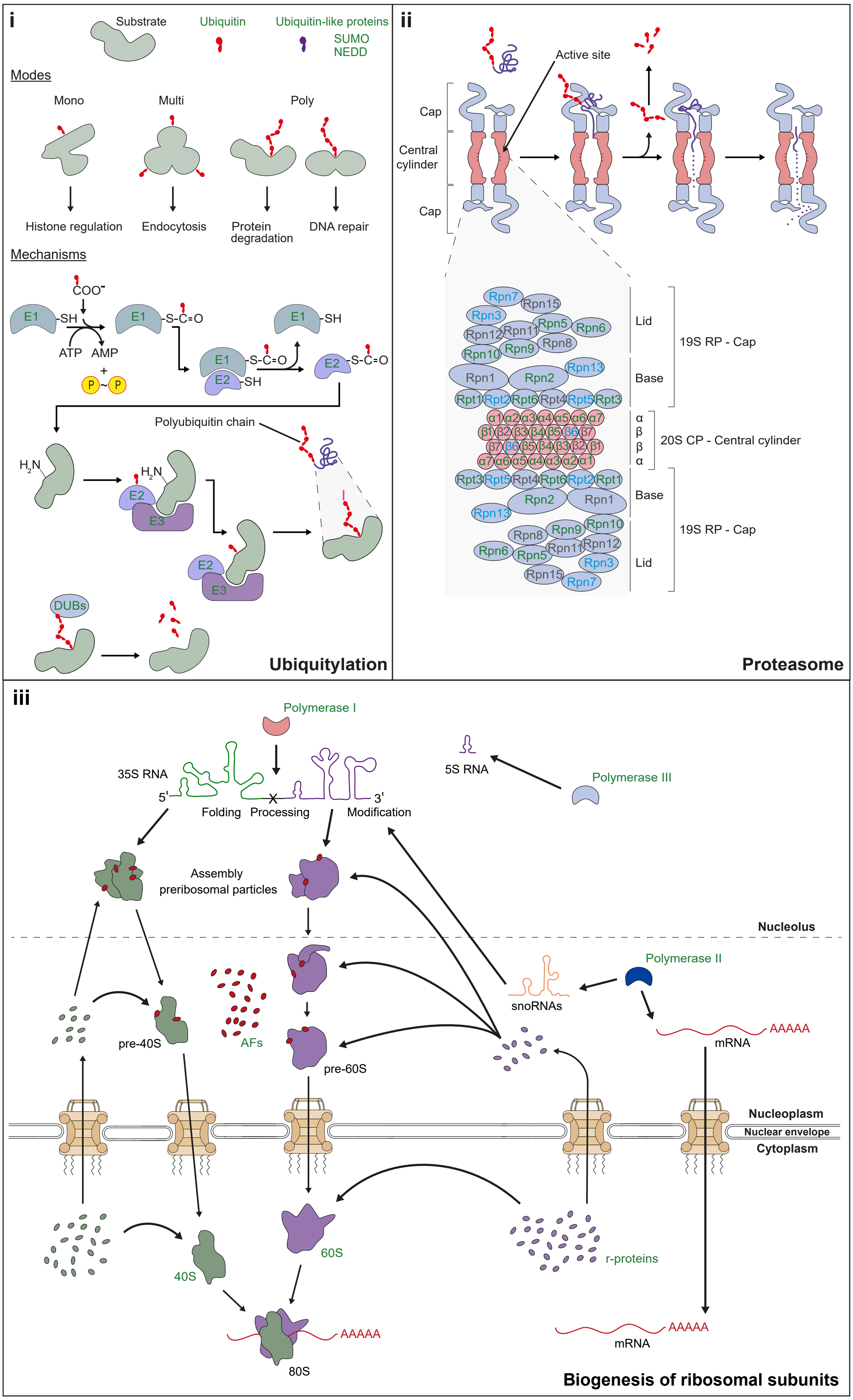

i
Substrate
Ubiquitin
Ubiquitin-like proteins
SUMO
NEDD
Modes
Mono
Multi
Poly
Histone regulation
Endocytosis
Protein degradation
DNA repair
Mechanisms
COO-
E1
-SH
ATP
AMP
+
P~P
E1
-S-C=O
E1
-SH
E1
-S-C=O
E2
-SH
E2
-S-C=O
Polyubiquitin chain
H2N
E2
E3
DUBs
Ubiquitylation
ii
Active site
Cap
Central cylinder
Cap
Rpn7
Rpn15
Rpn3
Rpn5
Rpn6
Rpn12
Rpn11
Rpn9
Rpn8
Rpn10
Rpn1
Rpn2
Rpn13
Rpt1
Rpt2
Rpt6
Rpt4
Rpt5
Rpt3
Lid
Base
19S RP - Cap
α
β
β
α
20S CP - Central cylinder
Base
19S RP - Cap
Lid
Proteasome
iii
Polymerase I
35S RNA
5'
3'
Folding
Processing
Modification
5S RNA
Polymerase III
Assembly preribosomal particles
Nucleolus
Polymerase II
snoRNAs
AAAAA
mRNA
pre-40S
AFs
pre-60S
Nucleoplasm
Nuclear envelope
Cytoplasm
40S
60S
r-proteins
80S
AAAAA
mRNA
Biogenesis of ribosomal subunits

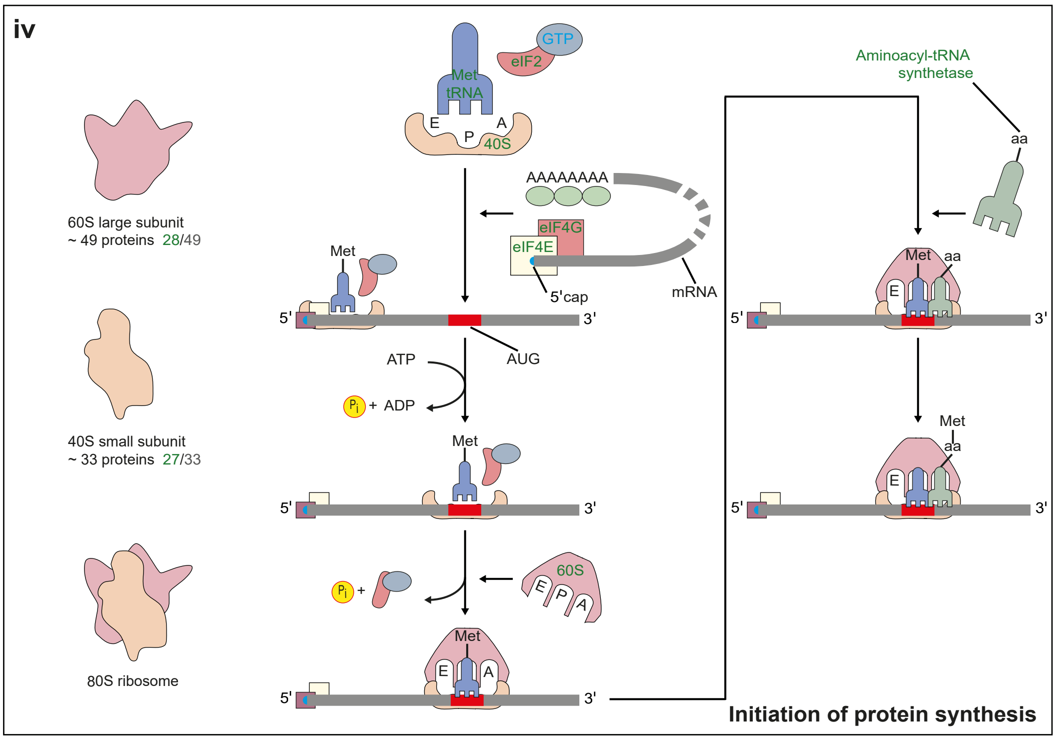

Fig. S5. Multi-organizational cellular processes (**i**) Ubiquitylation. Abbreviations: DUBs, deubiquitinating enzymes; NEDD, neddylin; SUMO, small ubiquitin-related modifier; UHRF1, E3 ubiquitin-protein ligase UHRF1. (Adapted from reference 29 with permission of the publisher.) (**ii**) Proteasome. Abbreviations: CP, core particle; RP, regulatory particle; Rpn, regulatory particle non-ATPase; Rpt, regulatory particle triple-ATPase. (Adapted from references 29 and 30 with permission of the publisher.) (**iii**) Biogenesis of translation-competent ribosomal subunits. Abbreviations: AF, assembly factor; r-proteins, ribosomal proteins; snoRNA, small nucleolar RNA. (Adapted from reference 31 with permission of the publisher.) (**iv**) Initiation of protein synthesis. Abbreviations: aa, aminoacyl-tRNA synthetase; eIF, eukaryotic initiation factor; GTP, guanosinetriphosphate; met-tRNA, methionine-tRNA synthetase. (Adapted from reference 29 with permission of the publisher.)

Multi-organizational cellular processes

(i) Ubiquitylation and neddylation. In eukaryotes, ubiquitylation yields diverse modifications of proteins serving essential functions ranging from signal transduction to cell division (32-34). These post-translational modifications can either be performed by the attach-ment of ubiquitin (Ub) or ubiquitin-like proteins (Ubl) to the targeted protein substrate (32, 34, 35). They are executed by the ubiquitin-conjugation system, which involves three consecutive enzymes: the ATP-dependent E1-ubiquitin activating enzyme binds Ub, followed by Ub-transfer to the E2-ubiquitin conjugating enzyme, from where Ub is either directly or via intermediary binding to E3-ubiquitin ligase transferred to the substrate (Fig. S5i) (36). Ubiquitylation of substrates can take on various proportions: transfer of a single Ub (monoubiquitylation), of multiple Ubs (multi-monoubiquitylation), or of mixed or branched Ub chains (polyubiquitylation) (32, 33). In *P. cordatum*, all three enzymes (E1, E2 and E3) could be identified by proteomics (geLC, shotgun) with higher abundances in the nuclear fraction. Moreover, a single Ub fragment and several Ubls as SUMO proteins (small ubiquitin-related modifier) could be identified with ~6-fold higher abundance in the nuclear fraction (shotgun). To reverse ubiquitylation, in response to e.g., environmental stress, deubiquitinating enzymes (DUBs) remove Ubs from the substrate (37). DUBs are classified into either metalloproteases or cysteine proteases (37), of which only the latter

could be assigned to nuclear functions in *P. cordatum*. The nuclear cysteine proteases Usp5/7/12/46/50 could be identified at low abundances in essentially all tested subcellular fractions; noteworthy Usp5/7 are apparently redox-sensitive (37). Taken together, the ubiquitylation machinery is present in *P. cordatum* as is also known for other dinoflagellates (38).

Neddylation represents a post-translational modification analogous to ubiquitylation and its widespread occurrence among eukaryotes suggests general cellular functions (39). However, the NEDD8 protein rather than Ubs is transferred to the protein substrate, likewise involving a tripartite enzyme cascade as described above for the ubiquitin-conjugation system (40). The NEDD8-specific E1-E3 were identified at ~8-fold higher abundance in the nuclear fraction (shotgun); as well as the accessory protein Cand1 (cullin-associated NEDD8-dissociated-protein). The COP9 signalosome complex plays a regulatory role in the interface between ubiquitylation, neddylation and protein degradation executed by the 26S proteasome (41). Notably, subunits 1 and 4 of this complex could be identified in the cellular fraction (shotgun) of *P. cordatum*.

(ii) Proteasome. Proteostasis requires misfolded, otherwise defective, or short-lived regulatory proteins to be removed from the intracellular protein pool (42). This is accomplished by the 26S proteasome (Fig. S5ii) recognizing such proteins due to their polyubiquitylation (30, 42). The large proteasome consists of two subcomplexes. First, the catalytic core particle (CP, synonym central cylinder, ~700 kDa) is composed of four seven-membered rings (two times each α1–α7 and β1–β7), which harbor the active site for protein degradation (42). Notably, almost all of the CP components could be detected in *P. cordatum* (geLC, shotgun), except for β6 (only predicted) and β2 and β7 (not encoded). Second, the regulatory particles (RPs, synonym cap, ~900 kDa), located on both sides of the CP, are composed of six Rpt proteins (ATPases) and thirteen Rpn proteins (42). The RPs recognize the polyubiquitylated proteins and unfold and translocate them to the CP (42). In *P. cordatum* Rpt4 and Rpn1/8/11/12/15 are apparently not encoded in its genome, whereas Rpt1/3/6 and Rpn2/5/6/9/10 were identified on the proteomic level with the remaining Rpt/Rpn proteins predicted only. Taken together, *P. cordatum* possess a fairly complete proteasome.

(iii) Biogenesis of translation-competent ribosomal subunits. Assembly and processing of translation-competent 40S and 60S ribosomal subunits mainly occurs in the nucleolus (31). Following transcription of manifold ribosomal genes, ribosome assembly factors and ribosomal proteins consolidate with the transcribed pre-rRNA to form pre-ribosomal particles (31). These further mature while traveling through the nucleoplasm to form via pre-40S and pre-60S the translation-competent 40S and 60S ribosomal subunits, which are exported through the NPCs into the cytoplasm (31). This complex process is orchestrated by ~200 different ribosomal assembly factors (AFs) and ~80 small nuclear RNAs (snoRNAs) (Fig. S5iii). Notably, only 2 AFs were predicted (1 identified) in the genome of *P. cordatum*, underscoring the lack of knowledge about ribosomal assembly in this dinoflagellate. In total, 27/33 of 28/46 predicted ribosomal proteins of the 40S/60S eukaryotic ribosomal subunits (43, 44) could be detected in this study. Moreover, 27 ribosomal proteins (30S and 50S) related to bacterial 70S ribosomes could be detected in *P. cordatum*, of which 3 could be identified as chloroplast-derived proteins. It remains presently unclear, whether the other ribosomal constituents are mitochondrial derived, originate from digested bacteria, or actually represent components of the dinoflagellate's ribosome. Detailed information of the identified ribosomes are summarized in Table S4.

(iv) Initiation of translation. Protein synthesis in eukaryotes is meditated by three interacting components: (i) a messenger RNA (mRNA), (ii) a methionyl-transfer RNA (Met-tRNA) and (iii) the completed 80S ribosome (29, 45). The first initiation step is the complex formation of the eukaryotic initiation factor 2 (eIF2) with the initiator Met-tRNA (Met-tRNAi) (29, 45). This initiator tRNA-methionine complex is further loaded into the small ribosomal subunit (1, 17). The association of several other eukaryotic initiation factors with the mRNA molecule allows the small ribosomal subunit to connect to the mRNA (29, 45).

Translation starts by arriving of the initiator tRNA-methionine complex at the translation start site (AUG) of the connected mRNA (29, 45). At this point all associated initiation factors release from this complex, which further allows the small and large ribosomal subunit to assemble (29, 45). The binding of aminoacyl-tRNA to the complex initiate the formation of the first peptide bond and protein synthesis can occur (29, 45). In *P. cordatum* 28 proteins could be identified as eukaryotic initiation factors, including subunits of eIF1, eIF2, eIF3, eIF4, eIF5, eIF6 and mRNA cap-binding proteins, 19 other proteins could be identified as elongation factors, comprising subunits 1, 2, 3 and 4 as well as and G, P, Ts and Tu. In addition, the protein methionine-tRNA ligase was detected twice, which is required for initiation of the mRNA translation through the Met-tRNA. Further, 55 different amino acid tRNA synthetases/ligases could be detected in the protein data of *P. cordatum*. Since a high number of proteins important for translation could be identified in the different datasets, one can assume, that the standard translation process occurs as known from other eukaryotes. Unlike to other eukaryotes, multi-codon genes and transcripts in *P. cordatum* indicate a polycistronic gene organization (26), as in bacteria and also found in other dinoflagellates (29, 46).

Further details to the multi-organizational cellular processes in *P. cordatum* are provided in Pcordatum_Proteomic_Tab4_protein data of nuclear processes.

References

1. Misteli T. 2020. The self-organizing genome: Principles of genome architecture and function. Cell J 183:28-45.
2. Hammond CM, Strømme CB, Huang H, Patel DJ, Groth A. 2017. Histone chaperone networks shaping chromatin function. Nat Rev Mol Cell Biol 18:141-158.
3. Bhat P, Honson D, Guttman M. 2021. Nuclear compartmentalization as a mechanism of quantitative control of gene expression. Nat Rev Mol Cell Biol 22:653-670.
4. Johnson A, O'Donnell M. 2005. Cellular DNA replicases: components and dynamics at the replication fork. Annu Rev Biochem 74:283-315.
5. Němečková A, Koláčková V, Vrána J, Doležel J, Hřibová E. 2020. DNA replication and chromosome positioning throughout the interphase in three-dimensional space of plant nuclei. J Exp Bot 71:6262-6272.
6. Kilchert C, Wittmann S, Vasiljeva L. 2016. The regulation and functions of the nuclear RNA exosome complex. Nat Rev Mol Cell Biol 17:227-239.
7. Wasmuth EV, Lima CD. 2012. Structure and activities of the eukaryotic RNA exosome. The Enzymes 31:53-75.
8. Van Hoof A, Frischmeyer PA, Dietz HC, Parker R. 2002. Exosome-mediated recognition and degradation of mRNAs lacking a termination codon. Science 295:2262-2264.
9. Carninci P, Sandelin A, Lenhard B, Katayama S, Shimokawa K, Ponjavic J, Semple CAM, Taylor MS, Engström PG, Frith MC, Forrest ARR, Alkema WB, Tan SL, Plessy C, Kodzius R, Ravasi T, Kasukawa T, Fukuda S, Kanamori-Katayama M, Kitazume Y, Kawaji H, Kai C, Nakamura M, Konno H, Nakano K, Mottagui-Tabar S, Arner P, Chesi A, Gustincich S, Persichetti F, Suzuki H, Grimmond SM, Wells CA, Orlando V, Wahlestedt C, Liu ET, Harbers M, Kawai J, Bajic VB, Hume DA, Hayashizaki Y. 2006. Genome-wide analysis of mammalian promoter architecture and evolution. Nat Genet 38:626-635.
10. Thomas MC, Chiang C-M. 2006. The general transcription machinery and general cofactors. Crit Rev Biochem Mol Biol 41:105-178.
11. Yokoyama A. 2019. RNA Polymerase II-dependent transcription initiated by selectivity factor 1: A central mechanism used by MLL fusion proteins in leukemic transformation. Front Genet 9:722.
12. Plaschka C, Larivière L, Wenzeck L, Seizl M, Hemann M, Tegunov D, Petrotchenko EV, Borchers CH, Baumeister W, Herzog F, Villa E, Cramer P. 2015. Architecture of the RNA polymerase II–Mediator core initiation complex. Nature 518:376-380.
13. Orphanides G, Lagrange T, Reinberg D. 1996. The general transcription factors of RNA polymerase II. Genes Dev 10:2657-2683.
14. Patel AB, Greber BJ, Nogales E. 2020. Recent insights into the structure of TFIID, its assembly, and its binding to core promoter. Curr Opin Struct Biol 61:17-24.
15. Giaquinto L, Curmi PM, Siddiqui KS, Poljak A, DeLong E, DasSarma S, Cavicchioli R. 2007. Structure and function of cold shock proteins in archaea. J Bacteriol 189:5738-5748.
16. Phadtare S, Severinov K. 2010. RNA remodeling and gene regulation by cold shock proteins. RNA Biol 7:788-95.

17. Sasaki K, Imai R. 2012. Pleiotropic roles of cold shock domain proteins in plants. Front Plant Sci 2:116.
18. Wahl MC, Will CL, Lührmann R. 2009. The spliceosome: design principles of a dynamic RNP machine. Cell 136:701-718.
19. Han J, Xiong J, Wang D, Fu XD. 2011. Pre-mRNA splicing: where and when in the nucleus. Trends Cell Biol 21:336-343.
20. Yoshida H, Park SY, Oda T, Akiyoshi T, Sato M, Shirouzu M, Tsuda K, Kuwasako K, Unzai S, Muto Y, Urano T, Obayashi E. 2015. A novel 3' splice site recognition by the two zinc fingers in the U2AF small subunit. Genes Dev 29:1649-1660.
21. Akey CW, Singh D, Ouch C, Echeverria I, Nudelman I, Varberg JM, Yu Z, Fang F, Shi Y, Wang J, Salzberg D, Song K, Xu C, Gumbart JC, Suslov S, Unruh J, Jaspersen SL, Chait BT, Sali A, Fernandez-Martinez J, Ludtke SJ, Villa E, Rout MP. 2022. Comprehensive structure and functional adaptations of the yeast nuclear pore complex. Cell 185:361-378.
22. Mosalaganti S, Obarska-Kosinska A, Siggel M, Taniguchi R, Turoňová B, Zimmerli CE, Buczak K, Schmidt FH, Margiotta E, Mackmull M-T, Hagen WJH, Hummer G, Kosinski J, Beck M. 2022. AI-based structure prediction empowers integrative structural analysis of human nuclear pores. Science 376:eabm9506.
23. Schuller AP, Wojtynek M, Mankus D, Tatli M, Kronenberg-Tenga R, Regmi SG, Dip PV, Lytton-Jean AKR, Brignole EJ, Dasso M, Weis K, Medalia O, Schwartz TU. 2021. The cellular environment shapes the nuclear pore complex architecture. Nature 598:667-671.
24. Fontana P, Dong Y, Pi X, Tong AB, Hecksel CW, Wang L, Fu TM, Bustamante C, Wu H. 2022. Structure of cytoplasmic ring of nuclear pore complex by integrative cryo-EM and AlphaFold. Science 376::eabm9326.
25. Miyamoto Y, Yamada K, Yoneda Y. 2016. Importin α: a key molecule in nuclear transport and non-transport functions. J Biochem 160:69-75.
26. Dougan KE, Deng Z-L, Wöhlbrand L, Reuse C, Bunk B, Chen Y, Hartlich J, Hiller K, John U, Kalvelage J, Mansky J, Neumann-Schaal M, Overmann J, Petersen J, Sanchez-Garcia S, Schmidt-Hohagen K, Shah S, Sproer C, Sztajer H, Wang H, Bhattacharya D, Rabus R, Jahn D, Chan CX, Wagner-Doebler I. 2022. Evolution of resilience against heat stress in a red-tide dinoflagellate. bioRxiv doi:10.1101/2022.07.25.501386:1-46.
27. Matsuura Y, Stewart M. 2005. Nup50/Npap60 function in nuclear protein import complex disassembly and importin recycling. EMBO J 24:3681-3689.
28. Grünwald D, Singer RH, Rout M. 2011. Nuclear export dynamics of RNA-protein complexes. Nature 475:333-341.
29. Alberts B, Johnson A, Lewis J, D. M, Raff M, Roberts K, Walter P. 2014. Molecular Biology of the Cell. Garland Publishing Inc. 6:1-1465.
30. Tanaka K. 2009. The proteasome: overview of structure and functions. Proc Jpn Acad Ser B Phys Biol Sci 85:12-36.
31. Baßler J, Hurt E. 2019. Eukaryotic ribosome assembly. Annu Rev Biochem 88:281-306.
32. Yau R, Rape M. 2016. The increasing complexity of the ubiquitin code. Nat Cell Biol 18:579-586.
33. Streich FC, Lima CD. 2014. Structural and functional insights to ubiquitin-like protein conjugation. Annu Rev Biophys 43:357-379.
34. Hochstrasser M. 2009. Origin and function of ubiquitin-like proteins. Nature 458:422-429.
35. van der Veen AG, Ploegh HL. 2012. Ubiquitin-like proteins. Annu Rev Biochem 81:323-357.
36. Liu W, Tang X, Qi X, Fu X, Ghimire S, Ma R, Li S, Zhang N, Si H. 2020. The ubiquitin conjugating enzyme: An important ubiquitin transfer platform in ubiquitin-proteasome system. Int J Mol Sci 21:2894.
37. Snyder NA, Silva GM. 2021. Deubiquitinating enzymes (DUBs): Regulation, homeostasis, and oxidative stress response. J Biol Chem 297: 101077.
38. Roy S, Morse D. 2013. Transcription and maturation of mRNA in dinoflagellates. Microorganisms 1:71-99.
39. Rabut G, Peter M. 2008. Function and regulation of protein neddylation. Protein modifications: beyond the usual suspects' review series. EMBO Rep 9:969-976.
40. Enchev RI, Schulman BA, Peter M. 2015. Protein neddylation: beyond cullin–RING ligases. Nat Rev Mol Cell Biol 16:30-44.
41. Cope GA, Deshaies RJ. 2003. COP9 signalosome: A multifunctional regulator of SCF and other cullin-based ubiquitin ligases. Cell 114:663-671.
42. Lasker K, Förster F, Bohn S, Walzthoeni T, Villa E, Unverdorben P, Beck F, Aebersold R, Sali A, Baumeister W. 2012. Molecular architecture of the 26S proteasome holocomplex determined by an integrative approach. Proc Natl Acad Sci USA 109:1380-1387.
43. de la Cruz J, Karbstein K, Woolford JL, Jr. 2015. Functions of ribosomal proteins in assembly of eukaryotic ribosomes in vivo. Annu Rev Biochem 84:93-129.

44. Woolford J. 2015. Assembly of ribosomes in eukaryotes. RNA (New York, NY) 21:766-768.
45. Merrick WC, Pavitt GD. 2018. Protein synthesis initiation in eukaryotic cells. Cold Spring Harb Perspect Biol 10:a033092.
46. Lin S, Zhang H, Zhuang Y, Tran B, Gill J. 2010. Spliced leader-based metatranscriptomic analyses lead to recognition of hidden genomic features in dinoflagellates. Proceedings of the National Academy of Sciences 107:20033-20038.
