# Supplementary material for: The enigmatic nucleus of the marine dinoflagellate Prorocentrum cordatum
Source: mSphere. 2023 Jun 26;8(4):e00038-23. doi: 10.1128/msphere.00038-23 (PMC10449503; doi:10.1128/msphere.00038-23)
Supplement: TABLE S1 — Literature data for comparison of P. cordatum genome size and chromosome numbers. [file msphere.00038-23-s0006.pdf]

Table S1. Literature data for comparison of *P. cordatum* genome size and chromosome numbers.

Organism	Median genome size [Gbp]	No. of chromosomes	Ploidy[a]	References[b]
**Protista**				
Prorocentrum cordatum	4.16	62	▲	Study object
Crypthecodinium cohnii	14	99	▲	(1-4)
Cryptosporidium parvum	0.01	8	▲	NCBI
Fugacium kawagutii (Symbiodinium kawagutii)	1.07	26	▲	(5, 6)
Giardia intestinalis	0.01	5	▲	NCBI
Guillardia theta	0.09	3	●	NCBI
Karenia brevis (Ptychodiscus brevis)	100	121	●	(1, 7, 8)
Oxyrrhis marina	0.06	55	▲	(1, 9, 10)
Plasmodium falciparum	0.02	14	●	NCBI
Plasmodium yoelii yoelii	0.02	14	●	NCBI
Prorocentrum micans	70	65	▲	(3, 4)
Symbiodinium microadriaticum	0.8	94	▲	(11)
Breviolum minutum	0.6	91	▲	(12)
Thalassiosira pseudonana	0.03	48	■	NCBI
**Animalia**				
Acropora millepora	0.43	28	■	NCBI,(13)
Adineta vaga	0.21	12	■	NCBI
Ambystoma mexicanum	28.21	28	■	NCBI
Anas platyrhynchos	1.19	68	■	NCBI,(14)
Argiope bruennichi	1.67	13	▲	NCBI,(15)
Callinectes sapidus	1.00	50	▲	NCBI
Canis lupus familiaris	2.34	78	■	NCBI
Carcharodon carcharias	3.92	82	■	NCBI,(16)
Chelonia mydas	2.13	56	■	NCBI,(17)
Clonorchis sinensis	0.55	14	■	NCBI
Cyprinus carpio	1.46	100	■	NCBI
Daphnia magna	0.15	10	▲	NCBI,(18)
Drosophila melanogaster	0.14	10	■	NCBI,(19)
Equus asinus	2.39	62	■	NCBI
Gallus gallus	1.05	78	■	NCBI
Heterodera glycines	0.14	18	■	NCBI,(20)
Homo sapiens	2.87	46	■	NCBI
Hormiphora californensis	0.11	26	■	NCBI,(21)
Lethenteron reissneri	1.06	72	▲	NCBI
Lytechinus pictus	1.00	19	▲	NCBI,(22)
Metaphire vulgaris	0.73	82	■	NCBI
Mus musculus	2.59	40	■	NCBI
Mytilus edulis	1.74	28	■	NCBI,(23)
Ornithorhynchus anatinus	1.92	52	■	NCBI
Strongyloides ratti	0.04	5	■	NCBI,(24)
Tachypleus gigas	1.83	14	▲	NCBI
Tapirus terrestris	2.57	80	■	NCBI,(25)
Thunnus albacares	0.79	48	■	NCBI,(26)
**Fungi**				
Antonospora locustae	0.003	17	▲	NCBI,(27)
Aspergillus nidulans	0.03	8	▲	NCBI,(28)
Bathycoccus prasinos	0.02	19	▲	NCBI
Neurospora crassa	0.04	7	▲	NCBI
Schizophyllum commune	0.04	11	▲	NCBI
**Plantae**				
Arabidopsis thaliana	0.12	5	▲	NCBI
Brachypodium distachyon	0.22	5	▲	NCBI
Ceratopteris richardii	5.85	39	▲	NCBI
Physcomitrium patens	0.47	27	▲	NCBI
Pinus taeda	22.1	12	▲	NCBI
Prunus persica	0.22	16	▲	NCBI
Zea mays	2.19	20	■	NCBI

[a]Ploidy symbols: triangle, unknown; circle, haploid; square, diploid.

[b]Further information on NCBI are provided in the reference list.

References

1. Spector DL. 1984. 4 - Dinoflagellate Nuclei, *In* Spector Dinoflagellates Academic Press, San Diego 107-147.
2. Kubai DF, Ris H. 1969. Division in the Dinoflagellate *Gyrodinium cohnii* (Schiller). A new type of nuclear reproduction. J Cell Biol 40:508-528.
3. Soyer-Gobillard MO, Dolan MF. 2015. Chromosomes of protists: The crucible of evolution. Int Microbiol 18:209-216.
4. Haapala OK, Soyer MO. 1973. Structure of dinoflagellate chromosomes. Nat New Biol 244:195-197.
5. Trench RK, Blank RJ. 1987. *Symbiodinium microadriaticum* Freudenthal, *S. goreauii* sp. nov., *S. kawagutii* sp. nov. and *S. pilosum* sp. nov.: *Gymnodinioid* dinoflagellate symbionts of marine invertebrates J Phycol 23:469-481.
6. Liu H, Stephens TG, González-Pech RA, Beltran VH, Lapeyre B, Bongaerts P, Cooke I, Aranda M, Bourne DG, Forêt S, Miller DJ, van Oppen MJH, Voolstra CR, Ragan MA, Chan CX. 2018. *Symbiodinium* genomes reveal adaptive evolution of functions related to coral-dinoflagellate symbiosis. Commun Biol 1:95.
7. Loper CL, Steidinger KA, Walker LM. 1980. A simple chromosome spread technique for unarmored dinoflagellates and implications of polyploidy in algal cultures. Trans Am Micros Soc 99:343-346.
8. Lidie K, Ryan J, Barbier M, Dolah F. 2005. Gene expression in Florida red tide dinoflagellate *Karenia brevis*: Analysis of an expressed sequence tag library and development of DNA microarray. J Mar Biotechnol 7:481-493.
9. Dodge J. 1963. Chromosome numbers in some marine dinoflagellates. Bot Mar 5:121-127.
10. Lowe CD, Mello LV, Samatar N, Martin LE, Montagnes DJS, Watts PC. 2011. The transcriptome of the novel dinoflagellate *Oxyrrhis marina* (Alveolata: Dinophyceae): response to salinity examined by 454 sequencing. BMC Genom 12:1-18.
11. Nand A, Zhan Y, Salazar OR, Aranda M, Voolstra CR, Dekker J. 2021. Genetic and spatial organization of the unusual chromosomes of the dinoflagellate *Symbiodinium microadriaticum*. Nat Genet 53:618-629.
12. Marinov GK, Trevino AE, Xiang T, Kundaje A, Grossman AR, Greenleaf WJ. 2021. Transcription-dependent domain-scale three-dimensional genome organization in the dinoflagellate *Breviolum minutum*. Nat Genet 53:613-617.
13. Wang S, Zhang L, Meyer E, Matz MV. 2009. Construction of a high-resolution genetic linkage map and comparative genome analysis for the reef-building coral *Acropora millepora*. Genome Biol 10:1-17.
14. Wójcik E, Smalec E. 2007. Description of the mallard duck (*Anas platyrhynchos*) karyotype. Folia Biol 55:115-120.
15. Sheffer MM, Hoppe A, Krehenwinkel H, Uhl G, Kuss AW, Jensen L, Jensen C, Gillespie RG, Hoff KJ, Prost S. 2021. Chromosome-level reference genome of the European wasp spider *Argiope bruennichi*: a resource for studies on range expansion and evolutionary adaptation. GigaScience 10:1-12.
16. Marra NJ, Stanhope MJ, Jue NK, Wang M, Sun Q, Pavinski Bitar P, Richards VP, Komissarov A, Rayko M, Kliver S, Stanhope BJ, Winkler C, O'Brien SJ, Antunes A, Jorgensen S, Shivji MS. 2019. White shark genome reveals ancient elasmobranch adaptations associated with wound healing and the maintenance of genome stability. Proc Natl Acad Sci USA 116:4446-4455.
17. Bickham JW, Bjorndal KA, Haiduk MW, Rainey WE. 1980. The karyotype and chromosomal banding patterns of the green turtle (*Chelonia mydas*). Copeia 1980:540-543.
18. Dukić M, Berner D, Roesti M, Haag CR, Ebert D. 2016. A high-density genetic map reveals variation in recombination rate across the genome of *Daphnia magna*. BMC Genet 17:137-150.
19. Kaufman TC. 2017. A short history and description of *Drosophila melanogaster* classical genetics: Chromosome aberrations, forward genetic screens, and the nature of mutations. J Genet 206:665-689.
20. Lian Y, Wei H, Wang J, Lei C, Li H, Li J, Wu Y, Wang S, Zhang H, Wang T, Du P, Guo J, Lu W. 2019. Chromosome-level reference genome of X12, a highly virulent race of the soybean cyst nematode *Heterodera glycines*. Mol Ecol Resour 19:1637-1646.
21. Schultz DT, Francis WR, McBroome JD, Christianson LM, Haddock SHD, Green RE. 2021. A chromosome-scale genome assembly and karyotype of the ctenophore *Hormiphora californensis*. G3: Genes Genomes Genetics 11:1-12.
22. Warner JF, Lord JW, Schreiter SA, Nesbit KT, Hamdoun A, Lyons DC. 2021. Chromosomal-level genome assembly of the painted sea *Urchin Lytechinus pictus*: A genetically enabled model system for cell biology and embryonic development. Genome Biol Evol 13:1-7.
23. Insua A, Labat JP, Thiriot Quievreux C. 1994. Comparative analysis of karyotypes and nucleolar organizer regions in different populations of *Mytilus trossulus*, *Mytilus edulis* and *Mytilus galloprovincialis* J Molluscan Stud 60:359-360.
24. Harvey SC, Viney ME. 2001. Sex determination in the parasitic nematode *Strongyloides ratti*. Genetics 158:1527-1533.
25. Houck ML, Kingswood SC, Kumamoto AT. 2000. Comparative cytogenetics of tapirs, genus tapirus (*Perissodactyla, tapiridae*). Cytogenet Cell Genet 89:110-115.
26. Lee Y-H, Yen T-B, Chen C-F, Tseng M-C. 2018. Variation in the karyotype, cytochrome b gene, and 5S rDNA of four thunnus (Perciformes, Scombridae) tunas. Zool Stud 57:1-13.
27. Chen L, Gao X, Li R, Zhang L, Huang R, Wang L, Song Y, Xing Z, Liu T, Nie X, Nie F, Hua S, Zhang Z, Wang F, Ma RZ, Zhang L. 2020. Complete genome of a unicellular parasite (*Antonospora locustae*) and transcriptional interactions with its host locust. Microb Genom 6:1-12.
28. Paoletti M, Seymour FA, Alcocer MJ, Kaur N, Calvo AM, Archer DB, Dyer PS. 2007. Mating type and the genetic basis of self-fertility in the model fungus *Aspergillus nidulans*. Curr Biol 17:1384-1389.

29. Schoch CL, Ciufo S, Domrachev M, Hotton CL, Kannan S, Khovanskaya R, Leipe D, McVeigh R, O'Neill K, Robbertse B, Sharma S, Soussov V, Sullivan JP, Sun L, Turner S, Karsch-Mizrachi I. 2020. NCBI Taxonomy: a comprehensive update on curation, resources and tools. Database (Oxford) 2020.

The NCBI taxonomy browser was used as baseline: Enter species name, go to table "Entrez records" and choose the database "Genome", choose subtree link, get genome and chromosome information (29); https://www.ncbi.nlm.nih.gov/taxonomy
