# Supplementary material for: The enigmatic nucleus of the marine dinoflagellate Prorocentrum cordatum
Source: mSphere. 2023 Jun 26;8(4):e00038-23. doi: 10.1128/msphere.00038-23 (PMC10449503; doi:10.1128/msphere.00038-23)
Supplement: TABLE S2 — Tested protocols for nuclei enrichment of P. cordatum. [file msphere.00038-23-s0007.pdf]

Table S2. Tested protocols for nuclei enrichment of *P. cordatum*.

Isolation buffer	pH	Cell disruption method and nuclei separation[a]	References
350 mM Sucrose, 10 mM $CaCl_2$, 5 mM $MgCl_2$, 0.5 mM PMSF, 0.02% Triton-X-100 (v/v)	8.2	5 ml-Dounce device, clearance unknown, 10x up/down, 3 times; (**A**) - filtration (8 µm)	(1)
350 mM Sucrose, 10 mM $CaCl_2$, 5 mM $MgCl_2$, 0.5 mM PMSF, 0.02% Triton-X-100 (v/v)	8.2	15 ml-Dounce device, clearance unknown, 10x up/down, 3 times; (**A**) - filtration (8 µm)	(1)
350 mM Sucrose, 10 mM $CaCl_2$, 5 mM $MgCl_2$, 0.5 mM PMSF, 0.02% Triton-X-100 (v/v)	8.2	French press, 200 psi; (**A**) - filtration (8 µm)	(1)
350 mM Sucrose, 10 mM $CaCl_2$, 5 mM $MgCl_2$, 0.5 mM PMSF, 0.02% Triton-X-100 (v/v)	8.2	French press, 400 psi; (**A**) - filtration (8 µm)	(1)
Synthetic ocean water (SOW)	8.2	Temperature, 4°C and 20°C; (**B**) - filtration (8 µm)	(2)
Tris-$MgCl_2$	7.5	Temperature, 4°C and 60°C, (**B**)	(2)
Tris-$MgCl_2$	7.5	Temperature, 4°C and 60°C, alternating, 2 times; (**B**)	(2)
Tris-$MgCl_2$	7.5	French press, 100 psi; (**A**)	-
Tris-$MgCl_2$	7.5	French press, 40 psi; (**A**)	-
1 M Sorbitol/mannitol (1:1); 4% cellulase	6.5	Enzyme, 15 h, 50°C; shaking with 1 mm silica beads, 30 sec; (**A**, **C**)	(3)
350 mM Sucrose, 10 mM $CaCl_2$, 5 mM $MgCl_2$	7.0	Enzyme, 45 min, 50°C; (**C**)	-
350 mM Sucrose, 10 mM $CaCl_2$, 5 mM $MgCl_2$	7.0	Enzyme, 45 min, 25°C; (**C**)	(4)
350 mM Sucrose, 10 mM $CaCl_2$, 5 mM $MgCl_2$	7.0	Enzyme, 45 min, 50°C; syringe, cannula (0.4 x 25 mm), in and out 4 times; (**A**)	-
350 mM Sucrose, 10 mM $CaCl_2$, 5 mM $MgCl_2$	7.0	Enzyme, 45 min, 25°C, syringe, cannula (0.4 x 25 mm), in and out 4 times; (**A**)	-
7 M NaCl, 0.0125% SDS; 0.34 M NaCl, 1 mM THAM; 15% cellulase	8.1	Osmotic pressure (high salt concentrations); (**C**) - Ficoll cushion	(4)
250 mM Sucrose, 1 mM EDTA, 20 mM Tris/HCl); 15% cellulase	7.4	Enzyme, 60 min, 350 rpm, 30°C; (**A**, **C**)	-
250 mM Sucrose, 1 mM EDTA, 20 mM Tris/HCl; 1.0 KU cellulase	7.4	Enzyme, 60 min, 100 rpm, 30°C, dark; dounce device, clearance B, 10x up/down, 3 times; (**A**, **C**)	-
250 mM Sucrose, 1 mM EDTA, 20 mM Tris/HCl; 1.0 KU cellulase	7.4	Enzyme, 60 min, 100 rpm, 30°C, dark; grinding kit, 2 min; (**A**, **C**)	-
250 mM Sucrose, 1 mM EDTA, 20 mM Tris/HCl; 1.0 KU cellulase	7.4	Enzyme, 60 min, 100 rpm, 30°C, dark; syringe, cannula (0.4 x 25 mm), in and out 10 times; (**A**, **C**)	-
250 mM Sucrose, 1 mM EDTA, 20 mM Tris/HCl	7.4	Sonication bath, 30 min; (**A**)	-

Isolation buffer	pH	Cell disruption method and nuclei separation[a]	References
250 mM Sucrose, 1 mM EDTA, 20 mM Tris/HCl	7.4	Sonication bath, 60 min; (**A**)	-
250 mM Sucrose, 1 mM EDTA, 20 mM Tris/HCl	7.4	Bead beating, 10 m/s, 10 sec; (**A**)	-
H_2O	7.4	Osmotic pressure; (**C**)	-
250 mM Sucrose, 1 mM EDTA, 20 mM Tris/HCl, 1.0 KU cellulase	7.4	Enzyme, 60 min, 100 rpm, 30°C, dark; french press, 200 psi; (**A**, **C**) - filtration (12 µm)	-
250 mM Sucrose, 1 mM EDTA, 20 mM Tris/HCl	7.4	Dounce device, clearance B, 10x up/down, 3 times, high cell density; (**A**)	-
250 mM Sucrose, 1 mM EDTA, 20 mM Tris/HCl, 1.0 KU cellulase	7.4	Enzyme, 60 min, 100 rpm, 30°C, dark; dounce device, clearance B, 10x up/down, 3 times, high cell density; (**A**, **C**)	-
10 mM Tricine, 1 mM EDTA, 1 mM PMSF	7.3	Swelling buffer osmotic pressure; dounce device, clearance B, 10x up/down, 3 times, high cell density; (**A**, **C**)	-
10 mM Tricine, 1 mM EDTA, 1 mM PMSF, 1% Triton-X-100	7.3	Swelling buffer osmotic pressure; dounce device, clearance B, 10x up/down, 3 times, high cell density; (**A**, **C**)	-
250 mM Sucrose, 1 mM EDTA, 20 mM Tris/HCl, 1% Triton-X-100	7.4	Incubation 5 min; (**C**)	-
250 mM Sucrose, 1 mM EDTA, 20 mM Tris/HCl, 0.5% Triton-X-100	7.4	Incubation 5 min; (**C**)	-
250 mM Sucrose, 1 mM EDTA, 20 mM Tris/HCl, 0.5% Triton-X-100, 0.5 KU cellulase	7.4	Enzyme, 60 min, 100 rpm, 30°C, dark; (**C**)	-
250 mM Sucrose, 1 mM EDTA, 20 mM Tris/HCl, 0.5% Triton-X-100, 0.5 KU cellulase	7.4	Enzyme, 60 min, 100 rpm, 30°C, dark; shaking, 10 min; (**A**, **C**)	-
250 mM Sucrose, 1 mM EDTA, 20 mM Tris/HCl, 1% SDS	7.4	Enzyme, 60 min, 100 rpm, 30°C, dark; shaking, 10 min; (**A**, **C**)	-
250 mM Sucrose, 1 mM EDTA, 20 mM Tris/HCl, 30% EtOH	7.4	EtOH-incubation; sonication lance, 4°C, 1 min, output 40%, duty cycle 50%; (**A**, **C**)	(5)
250 mM Sucrose, 1 mM EDTA, 20 mM Tris/HCl), 30% EtOH	7.4	EtOH-incubation; sonication lance, 4°C, 1 min, output 20%, duty cycle 50%; (**A**, **C**)	(5)
250 mM Sucrose, 1 mM EDTA, 20 mM Tris/HCl, 30% EtOH	7.4	EtOH-incubation; sonication lance, 4°C, 1 min, output 20%, duty cycle 50%, 2 times; (**A**, **C**)	(5)
250 mM Sucrose, 1 mM EDTA, 20 mM Tris/HCl, 30% EtOH	7.4	EtOH-incubation; bead beating without beads, 6 m/s, 10 sec; (**A**, **C**)	(5)
250 mM Sucrose, 1 mM EDTA, 20 mM Tris/HCl, 30% EtOH	7.4	EtOH-incubation; bead beating without beads, 6 m/s, 10 sec, 2 times; (**A**, **C**)	(5)
250 mM Sucrose, 1 mM EDTA, 20 mM Tris/HCl, 30% EtOH	7.4	EtOH-incubation; dounce device, clearance unknown; 20x up/down, fast; (**A**, **C**)	(5)
250 mM Sucrose, 1 mM EDTA, 20 mM Tris/HCl, 30% EtOH	7.4	EtOH-incubation; sonication lance, 4°C, 1 min, output 25%, duty cycle 25%; (**A**, **C**)	(5)
250 mM Sucrose, 1 mM EDTA, 20 mM Tris/HCl, 30% EtOH	7.4	EtOH-incubation; sonication lance, 4°C, 1 min, output 25%, duty cycle 25%; (**A**, **C**) - (i) sucrose-Percoll gradient 3-layer (18, 23, 40%); (ii) sucrose cushion (2.5 M)	(5, 6)

Isolation buffer	pH	Cell disruption method and nuclei separation[a]	References
250 mM Sucrose, 1 mM EDTA, 20 mM Tris/HCl, 30% EtOH	7.4	EtOH-incubation; sonication lance, 4°C, 1 min, output 25%, duty cycle 25%; (**A**, **C**) - (i) 3-layer sucrose-Percoll gradient (18, 23, 40%); (ii) continuous gradient (24-60%)	(5, 6)
250 mM Sucrose, 1 mM EDTA, 20 mM Tris/HCl, 30% EtOH	7.4	EtOH-incubation; sonication lance, 4°C, 1 min, output 25%, duty cycle 25%; (**A**, **C**) - (i) 3-layer sucrose-Percoll gradient (18, 23, 40%); (ii) 5-layer sucrose-Percoll gradient (24, 30, 36, 42, 48%)	(5, 6)

[a]Abbreviations: (**A**) physical (mechanical) disruption (**B**) physical (thermocyclic) disruption (**C**) reagent-based disruption.

References

1. Kato KH, Moriyama A, Huitorel P, Cosson J, Cachon M, Sato H. 1997. Isolation of the major basic nuclear protein and its localization on chromosomes of the dinoflagellate, Oxyrrhis marina. Biol Cell 89:43-52.
2. Wang D-Z, Dong H-P, Li C, Xie Z-X, Lin L, Hong H-S. 2011. Identification and characterization of cell wall proteins of a toxic dinoflagellate *Alexandrium catenella* using 2D DIGE and MALDI TOF-TOF mass spectrometry. Evid Based Complementary Altern Med 1-11:984080.
3. Aach HG, Bartsch S, Feyen V. 1978. Studies on *Chlorella* protoplasts. Planta 139:257-260.
4. Adamich M, Sweeney BM. 1976. The preparation and characterization of *Gonyaulax* spheroplasts. Planta 130:1-6.
5. Levi-Setti R, Gavrilov KL, Rizzo PJ. 2008. Divalent cation distribution in dinoflagellate chromosomes imaged by high-resolution ion probe mass spectrometry. Eur J Cell Biol 87:963-976.
6. Schikowsky C, Thal B, Braun HP, Eubel H. 2018. Sample preparation for analysis of the plant mitochondrial membrane proteome. Methods Mol Biol 1696:163-183.
