# Supplementary material for: The enigmatic nucleus of the marine dinoflagellate Prorocentrum cordatum
Source: mSphere. 2023 Jun 26;8(4):e00038-23. doi: 10.1128/msphere.00038-23 (PMC10449503; doi:10.1128/msphere.00038-23)
Supplement: TABLE S3 — Top ten of unknown proteins of P. cordatum. [file msphere.00038-23-s0008.pdf]

Table S3. Top ten of unknown proteins of *P. cordatum*. (**Top**) With the highest enrichment factor (EF) for the geLC approach. (**Bottom**) With the highest enrichment factor (EF) for the shotgun approach. Further details to Table S2 are provided in: Pcordatum_Proteomic_Tab3_prediction.xlsx

		Relative shares (%)				EF		
**Gene**	**Protein**	**geLC[a]**	**SG[a]**	**geLC[b]**	**SG[b]**	**geLC**	**SG**	**Prediction[c]**
**geLC**								
Pcordatum_s422_g10554	Hypothetical	0.229	0.293	0.002	0.015	145.9	18.9	0.2
Pcordatum_s1230_g21880	Hypothetical	0.108	0.273	0.005	0.030	23.0	9.0	0.2
Pcordatum_s6293_g56318	Hypothetical	0.126	0.015	0.005	0.001	22.9	16.4	0
Pcordatum_s13253_g76603	no hit	0.032	0.047	0.002	0.068	20.5	0.7	0
Pcordatum_s397_g10114	Hypothetical	0.076	0.224	0.004	0.009	19.5	24.6	0
Pcordatum_s8640_g65144	Hypothetical	0.098	0.005	0.005	0.013	17.8	0.4	0.4
Pcordatum_s9096_g66537	Hypothetical	0.051	0.022	0.003	n.d.	16.2	n.a.	0.5
Pcordatum_s998_g19079	Hypothetical	0.047	0.003	0.004	0.006	11.9	0.6	0.2
Pcordatum_s6147_g55677	Hypothetical	0.009	0.008	0.001	0.001	11.9	10.1	0
Pcordatum_s5289_g51565	Hypothetical	0.008	n.d.	0.001	n.d.	10.2	n.a.	0
**Shotgun**								
Pcordatum_s72_g2721	Hypothetical	0.01	0.04	n.d.	0.0005	n.a.	85.44	0.3
Pcordatum_s8940_g66049	Hypothetical	0.02	0.11	n.d.	0.0015	n.a.	72.38	0
Pcordatum_s12168_g74442	Hypothetical	0.02	0.21	n.d.	0.0042	n.a.	49.27	0.2
Pcordatum_s8396_g64320	Hypothetical	0.01	0.07	n.d.	0.0015	n.a.	46.27	0.4
Pcordatum_s6339_g56517	Hypothetical	0.06	0.02	0.016	0.0008	3.92	29.93	0
Pcordatum_s1191_g21429	Hypothetical	n.d.	0.10	n.d.	0.0037	n.a.	28.17	0.2
Pcordatum_s3418_g40721	no hit	n.d.	0.06	n.d.	0.0022	n.a.	25.02	0.2
Pcordatum_s20484_g85270	Hypothetical	n.d.	0.10	n.d.	0.0040	n.a.	25.01	0
Pcordatum_s397_g10114	Hypothetical	0.08	0.22	0.004	0.0091	19.45	24.64	0
Pcordatum_s908_g17945	No hit	n.d.	0.01	n.d.	0.0002	n.a.	23.22	0.4

[a]Nuclear fractions

[b]Cellular fractions

[c]Prediction result ranging from 0-1

Abbreviations: n.d., not detected; n.a., not available
