# Supplementary material for: The enigmatic nucleus of the marine dinoflagellate Prorocentrum cordatum
Source: mSphere. 2023 Jun 26;8(4):e00038-23. doi: 10.1128/msphere.00038-23 (PMC10449503; doi:10.1128/msphere.00038-23)
Supplement: TABLE S4 — Sequence-based identification of ribosomal subunits detected for P. cordatum. [file msphere.00038-23-s0009.pdf]

Table S4. Sequence-based identification of ribosomal subunits detected for *P. cordatum*. Chloroplast-derived proteins are indicated in green. Further details to **Table S4** are provided in: Pcordatum_Proteomic_Tab6_ribosomes.xlsx

No.	Gene *P. cordatum*	Organism	Taxonomic classification	Component	rSU proteins
**30S rSU (ribosomal subunit)**					
1	Pcordatum_s1647_g26264	*Chlamydia pneumoniae*	Bacteria(Chlamydiae)	Chromosome	30S-S1
2	Pcordatum_s763_g15956	*Staphylococcus haemolyticus*	Bacteria(Firmicutes)	Chromosome	30S-S1
3	Pcordatum_s4495_g47378	*Staphylococcus saprophyticus*	Bacteria(Firmicutes)	Chromosome	30S-S1
4	Pcordatum_s8976_g66169	*Bacillus subtilis*	Bacteria(Firmicutes)	Chromosome	30S-S1
5	Pcordatum_s1420_g23927	*Bacillus subtilis*	Bacteria(Firmicutes)	Chromosome	30S-S1
6	Pcordatum_s3905_g43819	*Bacillus subtilis*	Bacteria(Firmicutes)	Chromosome	30S-S1
7	Pcordatum_s10643_g70854	*Agrobacterium radiobacter*	Bacteria(Proteobacteria)	Chromosome	30S-S16
8	Pcordatum_s6675_g57914	*Methanococcus aeolicus*	Archaea(Euryarchaeota)	Chromosome	30S-S17
9	Pcordatum_s3280_g39778	*Metallosphaera sedula*	Archaea(TACK)	Chromosome	30S-S19
10	Pcordatum_s1536_g25144	*Phaeodactylum tricornutum*	Eukaryota(SAR-Stramenopiles)	Chloroplast	30S-S2
11	Pcordatum_s2679_g35444	*Gloeothece citriformis*	Bacteria(Cyanobacteria)	Chromosome	30S-S4
12	Pcordatum_s8091_g63307	*Methanocaldococcus jannaschii*	Archaea(Euryarchaeota)	Chromosome	30S-S6e
13	Pcordatum_s75_g2820	*Methanocaldococcus jannaschii*	Archaea(Euryarchaeota)	Chromosome	30S-S6e
14	Pcordatum_s6013_g55078	*Thermosynechococcus"vestitus*	Bacteria(Cyanobacteria)	Chromosome	30S-S9
**40S rSU (ribosomal subunit)**					
15	Pcordatum_s2166_g31187	*Rattus norvegicus*	Eukaryota(Opisthokonta-Metazoa)	Chromosome	40S-S10
16	Pcordatum_s1141_g20807	*Rattus norvegicus*	Eukaryota(Opisthokonta-Metazoa)	Chromosome	40S-S11
17	Pcordatum_s297_g8149	*Cyanophora paradoxa*	Plantae(Glaucoplantae)	Chromosome	40S-S12
18	Pcordatum_s3026_g38006	*Cyanophora paradoxa*	Plantae(Glaucoplantae)	Chromosome	40S-S12
19	Pcordatum_s1357_g23252	*Agaricus bisporus*	Eukaryota(Opisthokonta-Fungi)	Chromosome	40S-S13
20	Pcordatum_s13257_g76611	*Sus scrofa*	Eukaryota(Opisthokonta-Metazoa)	Chromosome	40S-S14
21	Pcordatum_s4321_g46332	*Arabidopsis thaliana*	Plantae(Streptophyta)	Chromosome	40S-S14-2
22	Pcordatum_s2367_g32987	*Picea mariana*	Plantae(Streptophyta)	Chromosome	40S-S15
23	Pcordatum_s2206_g31545	*Dictyostelium discoideum*	Eukaryota(Amoebozoa-Evosea)	Chromosome	40S-S15a
24	Pcordatum_s1194_g21468	*Dictyostelium discoideum*	Eukaryota(Amoebozoa-Evosea)	Chromosome	40S-S15a
25	Pcordatum_s569_g13076	*Gossypium hirsutum*	Plantae(Streptophyta)	Chromosome	40S-S16
26	Pcordatum_s12535_g75200	*Theileria parva*	Eukaryota(Sar-Alveolata)	Chromosome	40S-S17
27	Pcordatum_s3694_g42540	*Theileria parva*	Eukaryota(Sar-Alveolata)	Chromosome	40S-S17
28	Pcordatum_s12903_g75930	*Argopecten irradians*	Eukaryota(Opisthokonta-Metazoa)	Chromosome	40S-S18
29	Pcordatum_s2620_g34992	*Branchiostoma belcheri*	Eukaryota(Opisthokonta-Metazoa)	Chromosome	40S-S18
30	Pcordatum_s341_g9062	*Arabidopsis thaliana*	Plantae(Streptophyta)	Chromosome	40S-S19-2
31	Pcordatum_s5928_g54689	*Arabidopsis thaliana*	Plantae(Streptophyta)	Chromosome	40S-S19-2
32	Pcordatum_s13364_g76803	*Arabidopsis thaliana*	Plantae(Streptophyta)	Chromosome	40S-S19-2
33	Pcordatum_s4121_g45156	*Xenopus laevis*	Eukaryota(Opisthokonta-Metazoa)	Chromosome	40S-S20
34	Pcordatum_s280_g7816	*Cyanophora paradoxa*	Plantae(Glaucoplantae)	Chromosome	40S-S21
35	Pcordatum_s1556_g25356	*Spodoptera frugiperda*	Eukaryota(Opisthokonta-Metazoa)	Chromosome	40S-S23
36	Pcordatum_s2149_g31025	*Arabidopsis thaliana*	Plantae(Streptophyta)	Chromosome	40S-S24-2
37	Pcordatum_s1546_g25265	*Dictyostelium discoideum*	Eukaryota(Amoebozoa-Evosea)	Chromosome	40S-S25
38	Pcordatum_s1282_g22422	*Dictyostelium discoideum*	Eukaryota(Amoebozoa-Evosea)	Chromosome	40S-S25
39	Pcordatum_s15992_g81029	*Dictyostelium discoideum*	Eukaryota(Amoebozoa-Evosea)	Chromosome	40S-S25
40	Pcordatum_s255_g7297	*Octopus vulgaris*	Eukaryota(Opisthokonta-Metazoa)	Chromosome	40S-S27-2

**40S rSU (ribosomal subunit)**					
41	Pcordatum_s9444_g67613	*Arabidopsis thaliana*	Plantae(Streptophyta)	Chromosome	40S-S27-3
42	Pcordatum_s3878_g43641	*Arabidopsis thaliana*	Plantae(Streptophyta)	Chromosome	40S-S27-3
43	Pcordatum_s562_g12970	*Kluyveromyces marxianus*	Eukaryota(Opisthokonta-Fungi)	Chromosome	40S-S28
44	Pcordatum_s4793_g49038	*Kluyveromyces marxianus*	Eukaryota(Opisthokonta-Fungi)	Chromosome	40S-S28
45	Pcordatum_s1586_g25643	*Arabidopsis thaliana*	Plantae(Streptophyta)	Chromosome	40S-S3-1
46	Pcordatum_s10196_g69662	*Arabidopsis thaliana*	Plantae(Streptophyta)	Chromosome	40S-S3-2
47	Pcordatum_s2004_g29732	*Cryptosporidium hominis*	Eukaryota(Sar-Alveolata)	Chromosome	40S-S3a
48	Pcordatum_s15012_g79653	*Oryza sativa subsp. japonica*	Plantae(Streptophyta)	Chromosome	40S-S3a
49	Pcordatum_s1637_g26172	*Cricetulus griseus*	Eukaryota(Opisthokonta-Metazoa)	Chromosome	40S-S4
50	Pcordatum_s647_g14318	*Arabidopsis thaliana*	Plantae(Streptophyta)	Chromosome	40S-S5-2
51	Pcordatum_s6880_g58731	*Arabidopsis thaliana*	Plantae(Streptophyta)	Chromosome	40S-S5-2
52	Pcordatum_s3210_g39316	*Arabidopsis thaliana*	Plantae(Streptophyta)	Chromosome	40S-S5-2
53	Pcordatum_s5777_g53967	*Ictalurus punctatus*	Eukaryota(Opisthokonta-Metazoa)	Chromosome	40S-S6
54	Pcordatum_s3276_g39755	*Ictalurus punctatus*	Eukaryota(Opisthokonta-Metazoa)	Chromosome	40S-S6
55	Pcordatum_s453_g11134	*Avicennia marina*	Plantae(Streptophyta)	Chromosome	40S-S7
56	Pcordatum_s5280_g51516	*Avicennia marina*	Plantae(Streptophyta)	Chromosome	40S-S7
57	Pcordatum_s16353_g81497	*Avicennia marina*	Plantae(Streptophyta)	Chromosome	40S-S7
58	Pcordatum_s7026_g59338	*Avicennia marina*	Plantae(Streptophyta)	Chromosome	40S-S7
59	Pcordatum_s2158_g31107	*Theileria parva*	Eukaryota(Sar-Alveolata)	Chromosome	40S-S8
60	Pcordatum_s400_g10175	*Zea mays*	Plantae(Streptophyta)	Chromosome	40S-S8
61	Pcordatum_s482_g11652	*Zea mays*	Plantae(Streptophyta)	Chromosome	40S-S8
62	Pcordatum_s3236_g39475	*Arabidopsis thaliana*	Plantae(Streptophyta)	Chromosome	40S-S9-1
63	Pcordatum_s8023_g63082	*Toxoplasma gondii*	Eukaryota(Sar-Alveolata)	Chromosome	40S-SA
64	Pcordatum_s1835_g28145	*Plasmodium berghei*	Eukaryota(Sar-Alveolata)	Chromosome	40S-SA
65	Pcordatum_s120_g4131	*Plasmodium berghei*	Eukaryota(Sar-Alveolata)	Chromosome	40S-SA
**50S rSU (ribosomal subunit)**					
66	Pcordatum_s5310_g51666	*Synechococcus sp.*	Bacteria(Cyanobacteria)	Chromosome	50S-L10
67	Pcordatum_s371_g9630	*Trieres chinensis*	Eukaryota(SAR-Stramenopiles)	Chloroplast	50S-L12
68	Pcordatum_s2216_g31629	*Thermosynechococcus vestitus*	Bacteria(Cyanobacteria)	Chromosome	50S-L17
69	Pcordatum_s170_g5347	*Caldicellulosiruptor bescii*	Bacteria(Firmicutes)	Chromosome	50S-L19
70	Pcordatum_s2790_g36276	*Synechococcus sp.*	Bacteria(Cyanobacteria)	Chromosome	50S-L22
71	Pcordatum_s9178_g66778	*Geobacter sulfurreducens*	Bacteria (Proteobacteria)	Chromosome	50S-L23
72	Pcordatum_s671_g14644	*Trichodesmium erythraeum*	Bacteria(Cyanobacteria)	Chromosome	50S-L24
73	Pcordatum_s875_g17504	*Deinococcus radiodurans*	Bacteria(Deinococcos)	Chromosome	50S-L27
74	Pcordatum_s5182_g51006	*Thermosynechococcus vestitus*	Bacteria(Cyanobacteria)	Chromosome	50S-L28
75	Pcordatum_s2823_g36557	*Ruminiclostridium cellulolyticum*	Bacteria(Firmicutes)	Chromosome	50S-L3
76	Pcordatum_s6286_g56284	*Trichodesmium erythraeum*	Bacteria(Cyanobacteria)	Chromosome	50S-L4
77	Pcordatum_s11819_g73675	*Heterosigma akashiwo*	Eukaryota(SAR-Stramenopiles)	Chloroplast	50S-L5
78	Pcordatum_s541_g12614	*Persephonella marina*	Aquificae(Aquificae)	Chromosome	50S-L9
**60S rSU (ribosomal subunit)**					
79	Pcordatum_s3770_g43005	*Danio rerio*	Eukaryota(Opisthokonta-Metazoa)	Chromosome	60S-L10
80	Pcordatum_s178_g5535	*Danio rerio*	Eukaryota(Opisthokonta-Metazoa)	Chromosome	60S-L10
81	Pcordatum_s3560_g41670	*Oryza sativasubsp. japonica*	Plantae(Streptophyta)	Chromosome	60S-L10a
82	Pcordatum_s1291_g22510	*Toxocara canis*	Eukaryota(Opisthokonta-Metazoa)	Chromosome	60S-L11
83	Pcordatum_s3471_g41082	*Arabidopsis thaliana*	Plantae(Streptophyta)	Chromosome	60S-L11-1

**60S rSU (ribosomal subunit)**					
84	Pcordatum_s12445_g75006	*Arabidopsis thaliana*	Plantae(Streptophyta)	Chromosome	60S-L11-1
85	Pcordatum_s500_g11960	*Schizosaccharomyces pombe*	Eukaryota(Opisthokonta-Fungi)	Chromosome	60S-L11-B
86	Pcordatum_s17725_g83111	*Mus musculus*	Eukaryota(Opisthokonta-Metazoa)	Chromosome	60S-L12
87	Pcordatum_s10752_g71136	*Mus musculus*	Eukaryota(Opisthokonta-Metazoa)	Chromosome	60S-L12
88	Pcordatum_s942_g18399	*Brassica napus*	Plantae(Streptophyta)	Chromosome	60S-L13-2
89	Pcordatum_s1882_g28596	*Cyanophora paradoxa*	Plantae(Glaucoplantae)	Chromosome	60S-L13a
90	Pcordatum_s21934_g85774	*Cyanophora paradoxa*	Plantae(Glaucoplantae)	Chromosome	60S-L13a
91	Pcordatum_s114_g3966	*Lumbricus rubellus*	Eukaryota(Opisthokonta-Metazoa)	Chromosome	60S-L14
92	Pcordatum_s20565_g85311	*Lumbricus rubellus*	Eukaryota(Opisthokonta-Metazoa)	Chromosome	60S-L14
93	Pcordatum_s1505_g24814	*Pisum sativum*	Plantae(Streptophyta)	Chromosome	60S-L14
94	Pcordatum_s9717_g68365	*Pisum sativum*	Plantae(Streptophyta)	Chromosome	60S-L14
95	Pcordatum_s7199_g60020	*Petunia hybrida*	Plantae(Streptophyta)	Chromosome	60S-L15
96	Pcordatum_s2302_g32369	*Petunia hybrida*	Plantae(Streptophyta)	Chromosome	60S-L15
97	Pcordatum_s4156_g45371	*Rattus norvegicus*	Eukaryota(Opisthokonta-Metazoa)	Chromosome	60S-L17
98	Pcordatum_s7776_g62182	*Rattus norvegicus*	Eukaryota(Opisthokonta-Metazoa)	Chromosome	60S-L17
99	Pcordatum_s2618_g34977	*Arabidopsis thaliana*	Plantae(Streptophyta)	Chromosome	60S-L18-1
100	Pcordatum_s4787_g49005	*Arabidopsis thaliana*	Plantae(Streptophyta)	Chromosome	60S-L18-3
101	Pcordatum_s2333_g32656	*Castanea sativa*	Plantae(Streptophyta)	Chromosome	60S-L18a
102	Pcordatum_s10762_g71169	*Zea mays*	Plantae(Streptophyta)	Chromosome	60S-L19
103	Pcordatum_s4049_g44732	*Arabidopsis thaliana*	Plantae(Streptophyta)	Chromosome	60S-L19-2
104	Pcordatum_s232_g6772	*Saccharomyces cerevisiae*	Eukaryota(Opisthokonta-Fungi)	Chromosome	60S-L21-A
105	Pcordatum_s59_g2268	*Caenorhabditis elegans*	Eukaryota(Opisthokonta-Metazoa)	Chromosome	60S-L22
106	Pcordatum_s6344_g56541	*Dictyostelium discoideum*	Amoebozoa(Evosea)	Chromosome	60S-L22-1
107	Pcordatum_s11930_g73916	*Danio rerio*	Eukaryota(Opisthokonta-Metazoa)	Chromosome	60S-L23
108	Pcordatum_s7373_g60654	*Fritillaria agrestis*	Plantae(Streptophyta)	Chromosome	60S-L23A
109	Pcordatum_s19920_g84925	*Plutella xylostella*	Eukaryota(Opisthokonta-Metazoa)	Chromosome	60S-L24
110	Pcordatum_s5498_g52605	*Arabidopsis thaliana*	Plantae(Streptophyta)	Chromosome	60S-L26-1
111	Pcordatum_s390_g9993	*Saccharomyces cerevisiae*	Eukaryota(Opisthokonta-Fungi)	Chromosome	60S-L27-A
112	Pcordatum_s11958_g73977	*Arabidopsis thaliana*	Plantae(Streptophyta)	Chromosome	60S-L28-1
113	Pcordatum_s564_g12995	*Schizosaccharomyces pombe*	Eukaryota(Opisthokonta-Fungi)	Chromosome	60S-L28e
114	Pcordatum_s1433_g24048	*Bos taurus*	Eukaryota(Opisthokonta-Metazoa)	Chromosome	60S-L3
115	Pcordatum_s11972_g74009	*Bos taurus*	Eukaryota(Opisthokonta-Metazoa)	Chromosome	60S-L3
116	Pcordatum_s4513_g47486	*Bos taurus*	Eukaryota(Opisthokonta-Metazoa)	Chromosome	60S-L3
117	Pcordatum_s12165_g74436	*Bos taurus*	Eukaryota(Opisthokonta-Metazoa)	Chromosome	60S-L3
118	Pcordatum_s449_g11050	*Ophiophagus hannah*	Eukaryota(Opisthokonta-Metazoa)	Chromosome	60S-L30
119	Pcordatum_s3500_g41284	*Ophiophagus hannah*	Eukaryota(Opisthokonta-Metazoa)	Chromosome	60S-L30
120	Pcordatum_s1303_g22656	*Paralichthys olivaceus*	Eukaryota(Opisthokonta-Metazoa)	Chromosome	60S-L31
121	Pcordatum_s515_g12207	*Arabidopsis thaliana*	Plantae(Streptophyta)	Chromosome	60S-L35a-3
122	Pcordatum_s5619_g53229	*Arabidopsis thaliana*	Plantae(Streptophyta)	Chromosome	60S-L36-1
123	Pcordatum_s852_g17196	*Arabidopsis thaliana*	Plantae(Streptophyta)	Chromosome	60S-L36-1
124	Pcordatum_s2845_g36718	*Xenopus tropicalis*	Eukaryota(Opisthokonta-Metazoa)	Chromosome	60S-L4-1
125	Pcordatum_s695_g15002	*Arabidopsis thaliana*	Plantae(Streptophyta)	Chromosome	60S-L4-1
126	Pcordatum_s1511_g24874	*Arabidopsis thaliana*	Plantae(Streptophyta)	Chromosome	60S-L4-1
127	Pcordatum_s552_g12798	*Theileria parva*	Eukaryota(SAR-Alveolata)	Chromosome	60S-L5
128	Pcordatum_s24_g1072	*Theileria parva*	Eukaryota(SAR-Alveolata)	Chromosome	60S-L5

**60S rSU (ribosomal subunit)**					
129	Pcordatum_s5362_g51932	*Tetrahymena thermophila*	Eukaryota(SAR-Alveolata)	Chromosome	60S-L6
130	Pcordatum_s745_g15704	*Tetrahymena thermophila*	Eukaryota(SAR-Alveolata)	Chromosome	60S-L6
131	Pcordatum_s8540_g64825	*Tetrahymena thermophila*	Eukaryota(SAR-Alveolata)	Chromosome	60S-L6
132	Pcordatum_s12023_g74119	*Arabidopsis thaliana*	Plantae(Streptophyta)	Chromosome	60S-L7-2
133	Pcordatum_s1447_g24218	*Arabidopsis thaliana*	Plantae(Streptophyta)	Chromosome	60S-L7-3
134	Pcordatum_s383_g9869	*Arabidopsis thaliana*	Plantae(Streptophyta)	Chromosome	60S-L7-4
135	Pcordatum_s50_g1997	*Arabidopsis thaliana*	Plantae(Streptophyta)	Chromosome	60S-L7-4
136	Pcordatum_s510_g12136	*Arabidopsis thaliana*	Plantae(Streptophyta)	Chromosome	60S-L7-4
137	Pcordatum_s19032_g84311	*Tetrahymena thermophila*	Eukaryota(SAR-Alveolata)	Chromosome	60S-L7a
138	Pcordatum_s10972_g71704	*Tetrahymena thermophila*	Eukaryota(SAR-Alveolata)	Chromosome	60S-L7a
139	Pcordatum_s8627_g65103	*Tetrahymena thermophila*	Eukaryota(SAR-Alveolata)	Chromosome	60S-L7a
140	Pcordatum_s2277_g32150	*Oryza sativasubsp. japonica*	Plantae(Streptophyta)	Chromosome	60S-L7a-2
141	Pcordatum_s4537_g47624	*Drosophila melanogaster*	Eukaryota(Opisthokonta-Metazoa)	Chromosome	60S-L8
142	Pcordatum_s771_g16071	*Oryza sativasubsp. japonica*	Plantae(Streptophyta)	Chromosome	60S-L9
143	Pcordatum_s406_g10287	*Oryza sativasubsp. japonica*	Plantae(Streptophyta)	Chromosome	60S-L9
